# Bacterial Community Development in Experimental Gingivitis

**DOI:** 10.1371/journal.pone.0071227

**Published:** 2013-08-14

**Authors:** James O. Kistler, Veronica Booth, David J. Bradshaw, William G. Wade

**Affiliations:** 1 Microbiology Unit, King's College London Dental Institute, London, United Kingdom; 2 Department of Periodontology, King's College London Dental Institute, London, United Kingdom; 3 GlaxoSmithKline Consumer Healthcare, Weybridge, Surrey, United Kingdom; University of Toronto, Canada

## Abstract

Current knowledge of the microbial composition of dental plaque in early gingivitis is based largely on microscopy and cultural methods, which do not provide a comprehensive description of oral microbial communities. This study used 454-pyrosequencing of the V1–V3 region of 16S rRNA genes (approximately 500 bp), and bacterial culture, to characterize the composition of plaque during the transition from periodontal health to gingivitis. A total of 20 healthy volunteers abstained from oral hygiene for two weeks, allowing plaque to accumulate and gingivitis to develop. Plaque samples were analyzed at baseline, and after one and two weeks. In addition, plaque samples from 20 chronic periodontitis patients were analyzed for cross-sectional comparison to the experimental gingivitis cohort. All of the healthy volunteers developed gingivitis after two weeks. Pyrosequencing yielded a final total of 344 267 sequences after filtering, with a mean length of 354 bases, that were clustered into an average of 299 species-level Operational Taxonomic Units (OTUs) per sample. Principal coordinates analysis (PCoA) plots revealed significant shifts in the bacterial community structure of plaque as gingivitis was induced, and community diversity increased significantly after two weeks. Changes in the relative abundance of OTUs during the transition from health to gingivitis were correlated to bleeding on probing (BoP) scores and resulted in the identification of new health- and gingivitis-associated taxa. Comparison of the healthy volunteers to the periodontitis patients also confirmed the association of a number of putative periodontal pathogens with chronic periodontitis. Taxa associated with gingivitis included *Fusobacterium nucleatum* subsp. *polymorphum*, *Lachnospiraceae* [G-2] sp. HOT100, *Lautropia* sp. HOTA94, and *Prevotella oulorum,* whilst *Rothia dentocariosa* was associated with periodontal health. Further study of these taxa is warranted and may lead to new therapeutic approaches to prevent periodontal disease.

## Introduction

Gingivitis is a reversible form of periodontal disease characterized by inflammation of the gingivae in response to a mature dental plaque biofilm. In susceptible individuals persistent gingivitis may lead to chronic periodontitis [1], which causes irreversible destruction of periodontal tissue. Currently, the major means of prevention are oral hygiene practices such as tooth brushing, interdental cleaning and the use of antimicrobial mouth rinses. Many individuals do not practice oral hygiene to a standard sufficient to prevent gingivitis and alternative preventive strategies are therefore desirable. Recently, there has been interest in the potential for using probiotics or prebiotics that aim to promote periodontal health by maintaining plaque in a health-associated state [2]–[4]. However, a more comprehensive knowledge of the bacterial composition of plaque in health, and the changes that occur during the initial stages of gingivitis are first required. The microbiota associated with chronic periodontitis has been investigated in more depth and was recently the subject of an extensive review [5].

The essential role of plaque in gingivitis was first shown using an ‘experimental gingivitis’ model [6], [7]. Using microscopy, the investigators noted changes in the predominant bacterial morphotypes present in plaque during the transition from health to gingivitis. In particular, they reported that early plaque in health consisted of a relatively simple bacterial community dominated by Gram-positive cocci and rods. As plaque matured, and gingivitis developed, the communities became increasingly complex with higher proportions of Gram-negative rods, fusiforms, filaments, spirilla and spirochetes. Later experimental gingivitis studies using culture confirmed these findings and provided more information regarding the specific bacterial species present in plaque [8]–[10]. It has been estimated, however, that approximately half of the bacteria found in the oral cavity have not been, or cannot be, cultivated in the laboratory [11]. Therefore, culture studies alone could not provide a comprehensive description of the microbiota in experimental gingivitis.

The introduction of culture-independent molecular methods to identify the bacteria present in complex samples, such as those based on cloning and Sanger sequencing of 16 S ribosomal RNA genes, has greatly expanded our knowledge of oral bacterial communities in health and disease [12]. Aas et al. [13] used this approach to characterize the bacterial communities at nine different oral sites in five healthy individuals and detected between 34 and 72 different species-level phylotypes per individual. The authors found that particular phylotypes showed site- and subject-specificity, while others such as *Streptococcus mitis* and *Granulicatella adiacens* were detected in the majority of the subjects and sites sampled. The so-called ‘red complex’ putative periodontal pathogens (*Porphyromonas gingivalis*, *Tannerella forsythia* and *Treponema denticola*), previously associated with chronic periodontitis, and each other, on the basis of checkerboard DNA-DNA hybridization [14], were not detected. Interestingly, though, a similar later investigation of the microbiota in 10 healthy individuals detected all three ‘red complex’ species at low numbers [15]. No previous reported studies have used 16S rRNA gene cloning and sequencing to characterize the microbiota in experimental gingivitis.

Whilst 16S rRNA gene cloning and Sanger sequencing has undoubtedly been useful, it suffers from being relatively low-throughput and researchers have typically sequenced a limited numbers of clones per sample (often ≤100) [13], [16]. In recent years, next-generation sequencing technologies have led to significant improvements in the depth and scale of 16S rRNA gene sequencing studies [17]. Using 454-pyrosequencing of 16S rRNA genes, Griffen [18] et al. and Abusleme et al. [19] recently compared the subgingival bacterial communities of chronic periodontitis patients to those of healthy individuals. In both cases the authors elucidated significant differences between cohorts in terms of the membership and structure of their subgingival communities and reported a higher bacterial diversity in disease. In addition, the authors revealed associations between specific bacterial taxa and disease. The ‘red complex’ species were associated with disease in both studies. Interestingly, however, they also identified a range of additional species that showed associations with chronic periodontitis. *Filifactor alocis* and *Treponema medium* were among those taxa most strongly associated with disease in both studies. One 454-pyrosequencing study examined bacterial community differences in the saliva and plaque of three healthy individuals and three individuals with gingivitis [20]. The communities in plaque, but not in saliva, differed significantly between health and gingivitis and a number of species-level OTUs in plaque were enriched or reduced in the individuals with gingivitis versus healthy individuals. Many of the OTUs associated with gingivitis were identified as members of the genera *Leptotrichia* and *Selenomonas*, although species-level assignments were not provided in most cases. One limitation of this and other cross-sectional studies though, is that large inter-individual variation, particularly at the species level, has been found in the oral microbiome [21]–[23].

This study aimed to use the longitudinal experimental gingivitis model, high-throughput 16S rRNA pyrosequencing and non-selective culture methods to comprehensively characterize the bacterial communities in plaque during the transition from health to experimentally-induced gingivitis. In addition, plaque samples from a group of patients with severe periodontitis were pyrosequenced as a cross-sectional comparison to the healthy cohort. The analyses sought to identify specific bacterial taxa associated with periodontal health and the initial stages of gingivitis.

## Materials and Methods

### Subject recruitment

Ethical approval for the study was granted by the South East London Research Ethics Committee 1 (formerly Guy's REC) and informed consent was obtained from all individuals who participated. All patients and subjects enrolled in the study had at least 20 teeth and were systemically healthy with no history of antibiotic use for at least three months prior to the study. Pregnant women, current smokers or individuals who quit smoking within the previous five years were not enrolled.

#### Periodontally healthy volunteers

Recruitment was made from clinical staff within the King's College London Dental Institute. All subjects recruited had no evidence of periodontitis and already had only minimal gingival inflammation with no need for professional intervention. The BPE (Basic Periodontal Examination) was used to screen potential subjects. Subjects had no BPE score greater than two in any of the sextants, no probing attachment loss, fewer than 15% sites bleeding after probing, and no evidence of gingival recession. All clinical examinations were undertaken by the same dentally qualified clinician.

#### Patients with chronic periodontitis

Patients were enrolled from those referred to the Department of Periodontology at Guy's and St Thomas' Foundation Trust for treatment. All patients had a minimum of 20 teeth and were diagnosed with severe chronic periodontitis [24]. Each patient had at least six teeth with probing depths of ≥6 mm and bone loss. Patients had a minimum of 20 teeth and at least six teeth with probing depths of ≥6 mm.

### Experimental gingivitis

**Figure 1:**
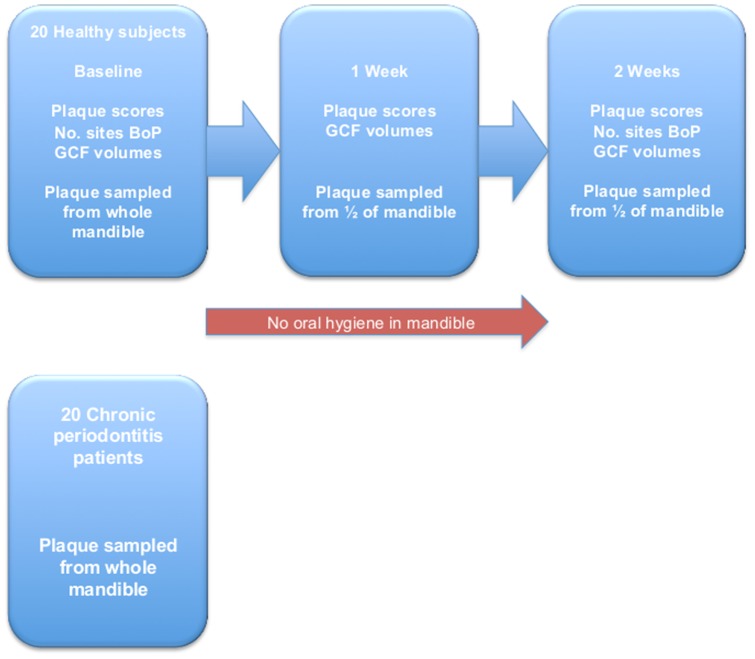
Study design.

A summary of the experimental gingivitis study design is shown in Figure 1. Subjects were instructed to abstain from all methods of tooth cleaning in the mandible for two weeks. A soft acrylic stent was made to cover the mandibular teeth whilst brushing the maxillary teeth and was removed immediately after brushing. The soft stent could be easily inserted and removed without disturbance of the developing plaque whilst ensuring subjects could not brush their mandibular teeth and thus disrupt plaque formation. At baseline and after two weeks six sites/tooth were probed and bleeding on probing (BoP) was assessed. Probing after one week of plaque accumulation was avoided since bleeding in the crevice might have influenced the developing biofilm. However, at all time points, non-invasive samples of gingival crevicular fluid (GCF) were collected before clinical measurements or plaque sampling as the volume of GCF has been shown to increase before the development of clinically evident inflammation eg. BoP. The number of sites with clearly visible plaque (Silness and Loe plaque index = 2 [25]) were recorded. After two weeks all subjects had their teeth polished and they resumed normal oral hygiene practices.

### Sample collection

#### Experimental gingivitis

At baseline, after collection of GCF and clinical measurements, plaque samples were collected using a sterile curette from all the mandibular teeth in the healthy subjects with the exception of the third molars. Plaque samples were collected using a sterile curette from just above the gingival margin and from the gingival crevices into 1 ml of sterile 0.1x Tris-EDTA and plaque from all sites within the patient was pooled. The same method of collection was used throughout the study to ensure that samples were of a comparable nature. GCF was collected from the mesiobuccal sites of 12 mandibular teeth on periopaper strips and the volume of fluid estimated using a Periotron 8000. Samples of plaque and GCF were collected again after one and two weeks of plaque accumulation. Plaque was collected from teeth on one half of the mandible after one week and the other half after two weeks.

#### Chronic periodontitis

Superficial plaque samples were collected in the same way as those from the healthy subjects. For 14 of the 20 patients, separate samples of subgingival plaque were collected by inserting a curette to the full depth of pockets >6 mm after the superficial plaque had been collected. These sites were selected on the basis of pocket depth to represent a distinctly different microbial habitat from the superficial samples and the specific sites selected varied between individuals depending on the pattern of their disease. The clinical condition of the patients was so different from the group of healthy volunteers it was impossible to blind the examiner to which group the individuals came from. However, the use of a sampling technique that removed plaque from around the gingival margins and to the depth of a healthy crevice in both the experimental gingivitis and periodontitis groups standardized the physical environment from which the superficial plaque samples were collected. The superficial plaque from the healthy volunteers and periodontitis patients was used for inter-group comparison and an additional comparison was made between the superficial and subgingival samples within the patients.

### DNA extraction

DNA was extracted from the samples using the GenElute Bacterial DNA Extraction Kit (Sigma-Aldrich). Extractions were carried out following the manufacturer's instructions with an additional lysis step to increase the recovery of Gram-positive bacterial DNA: samples were incubated with a 45 mg/ml lysozyme solution at 37°C for 30 minutes. This protocol has been shown to be effective for the extraction of DNA from mock communities comprised of a mixture of Gram-positive and Gram-negative oral bacterial species (unpublished data). Extracted DNA was stored at −70°C until further processing.

### 16S rRNA gene PCR and 454 pyrosequencing

An approximately 500 bp region of the 16 S rRNA gene (covering V1–V3) was PCR-amplified from extracted DNA samples using composite fusion primers comprising universal 16 S primers (27FYM [26] and 519R [27]) along with Roche GS-FLX Titanium Series adapter sequences (A & B) for 454 pyrosequencing using the Lib-L emPCR method. Previously described unique 12 base error-correcting Golay barcode sequences [28] were incorporated into the forward primers (5′-CCATCTCATCCCTGCGTGTCTCCGACTCAG-NNNNNNNNNNNN-AGAGTTTGATYMTGGCTCAG-3′) to enable pooling of samples in the same sequencing run. The appropriate barcoded A-27FYM and the B-519R (5′-CCTATCCCCTGTGTGCCTTGGCAGTCTCAG-GWATTACCGCGGCKGCTG-3′) primers were used in PCRs with Extensor Hi-Fidelity PCR Mastermix (Thermo Scientific). There was an initial denaturation step of 5 mins at 95°C followed by 25 cycles of 95°C for 45 s, 53°C for 45 s, 72°C for 1 m 30 s and a final extension of 72°C for 15 mins. PCR amplicons were subsequently purified using the QIAquick PCR purification kit (Qiagen) following the manufacturer's instructions. The size and purity of the amplicons was checked using the Agilent DNA 1000 kit and the Agilent 2100 Bioanalyzer. The amplicons were quantitated by means of a fluorometric assay using the Quant-iT Picogreen fluorescent nucleic acid stain (Invitrogen) and then pooled at equimolar concentrations (1×10^9^ molecules/µl). emPCR and undirectional sequencing of the libraries was performed using the Lib-L kit and Roche 454 GS-FLX Titanium sequencer by the Centre for Haemato-Oncology, Barts Cancer Institute, Queen Mary University of London, London, UK.

### Sequence analysis

Pre-processing and analysis of sequences was carried out using the mothur analysis suite v 1.26.0 [29] based on the Schloss standardized operating procedure (SOP) [30]. Sequences were first de-noised using the AmpliconNoise algorithm [31] as implemented by mothur. After de-noising, any sequences that were less than 350 bp in length and/or had one of the following: >2 mismatches in the primer, >1 mismatch in barcode regions and homopolymers of >8 bases, were removed from the dataset. The remaining sequences were trimmed to remove the primers and barcodes and aligned to the SILVA 16S rRNA reference alignment [32]. The UChime algorithm [33] was used to identify and remove chimeric sequences. Sequences were identified by BLAST against the Human Oral Microbiome Database (HOMD) [34] at ≥98.5% sequence identity. Additionally, sequences were clustered into Operational Taxonomic Units (OTUs) at a sequence dissimilarity distance of 0.015 using an average neighbour algorithm and then classified using a Naïve Bayesian classifier with the HOMD v 10.1 reference dataset. Where necessary, possible alternatives for the species identifications were given. A previously described method was used to distinguish the commensal mitis-group streptococci from the closely related pathogen *Streptococcus pneumoniae* [35]. Good's non-parametric coverage estimator [36] was used to assess the extent of sampling of communities. Diversity of the communities was calculated using Simpson's inverse diversity index [37] and the total richness of the communities was estimated using Chao1[38] and CatchAll [39]. The Jaccard Index and the thetaYC metric [40] were used to generate distance matrices from sub-sampled sequence libraries (equal to that of the library with the fewest sequences), which were visualized as dendrograms and PCoA (Principal Coordinates Analysis) plots. Three-dimensional PCoA plots were generated in R (r-project.org) using the rgl package. The β diversity of communities was also compared based on their phylogenetic relatedness. For this, a neighbour-joining tree of the sequences constructed with Clearcut [41] was analyzed by unweighted and weighted UniFrac metrics [42] as implemented by mothur and visualized as above.

### Statistical analysis

The non-parametric Friedman and Wilcoxon-signed rank tests were used to test for the significance of differences in the OTU richness and diversity of samples from the different time points of experimental gingivitis. A Bonferroni correction for multiple comparisons was applied to the alpha value for pairwise comparisons. Analysis of Molecular Variance (AMOVA) [43] was performed in mothur to determine if clustering patterns seen in the PCoA plots were statistically supported by differences in the distance matrices. A Bonferroni correction for multiple comparisons was applied to the alpha value when comparing the time points of experimental gingivitis. Parsimony [44], as implemented by mothur, was used to determine if clustering in the dendrograms was significant. Associations of OTUs with time points of experimental gingivitis and BoP scores were detected using Multivariate Association with Linear Models (MaAsLin) [45]. Linear Discriminant Analysis Effect Size (LEfSe) [46] was used to detect significant differences in the relative abundances of OTUs between healthy and chronic periodontitis cohorts. The alpha values in LEfSe were set to 0.05 and an LDA threshold of 2.0 was applied. Two-sample *t*-tests and paired *t*-tests were performed in R to determine if differences in the relative abundances of phyla between the healthy and periodontitis cohort and between time points of experimental gingivitis were statistically significant. A Bonferroni correction for multiple comparisons was applied to the alpha values. Dichotomous plaque and bleeding scores were expressed as a percentage of the number of assessed sites. Percentage plaque scores were first analyzed using the Friedman test for non-parametric data to establish whether there was a statistically significant difference in the amount of plaque over the three time points and this was followed by comparison between time points using Wilcoxon sign-rank tests. Since percentage bleeding scores were only measured at two time points they were analyzed using a Wilcoxon sign-rank test. The volume of GCF was a continuous, normally distributed variable and was therefore first analyzed using a repeated measures analysis of variance and subsequently comparison between individual time points assessed using paired t-tests.

### Cultural analysis

For ten healthy subjects, baseline and two week plaque samples were cultured on non-selective media: First, 500μl of a 0.1x TE suspension containing plaque was sonicated for 20 s. Ten-fold serial dilutions of the sonicated samples (up to 10^5^) were made in pre-reduced transport medium and inoculated onto Blood Agar Base no. 2+5% v/v horse blood (BA) and Fastidious Anaerobe Agar +5% v/v horse blood (FAA) in triplicate. BA plates were incubated for 4 days in 5% C0_2_ + air at 37°C, whilst FAA plates were incubated for 10 days in an anaerobic cabinet with an atmosphere of 80% N_2_, 10% H_2_ and 10% C0_2_ at 37°C. After incubation, the plates were counted and 96 colonies (48 from BA and 48 from FAA) were subcultured at random [47]. Isolates were identified by 16S rRNA gene sequence analysis [48] and analyzed using mothur as described above.

### Investigation of potentially novel oral taxa

Phylotypes which did not match taxa in HOMD (<98.5% sequence identity) and were present in at least two individuals were investigated further. Specific forward primers were designed from the 454 sequences and used to amplify the 16 S rRNA gene of the target phylotype in combination with the primer 1492R. PCR amplicons of the correct size were subsequently cloned into *Escherichia. coli* using the TOPO cloning kit (Invitrogen, UK) and sequenced as described previously [48].

## Results

### Clinical results

The experimental gingivitis cohort comprised 16 females and four males with a mean age of 28.1 (± 2.1). 19 of the subjects completed the study. One subject withdrew from the study because of a chest infection. No other adverse affects or complications were reported. After two weeks all subjects had developed thick clearly visible plaque on most tooth surfaces. They had significantly increased gingival bleeding on probing (BoP) scores (Table 1) and the mean volume of gingival crevicular fluid (GCF) increased significantly after one and two weeks. A total of 11 female and nine male chronic periodontitis patients with a mean age of 48.5 (± 9) were sampled. A two-sample *t*-test showed that periodontitis patients were significantly older than the experimental gingivitis group. The mean number of teeth per patient was 28.1 (±2.8) whilst the number of teeth per patient with pocket depths ≥6 mm was 11.9 (±5.9). In 14 of the patients, subgingival plaque samples from deep pockets were obtained as an additional comparison to the plaque samples from around the gingival margin.

**Table 1:** Summary of clinical parameters of subjects during experimental gingivitis.

Time point	GCF volume (μl)	% of sites with Bleeding on Probing (BoP).	% of sites with visible plaque.
	Mean (±SD)	Median (IQR)	Median (IQR)
Baseline	6.82 (±2.01)	7.04 (4.8–10.7)	6.7 (1.8–10.0)
1 Week	8.22 (±1.94)	N/A	62.4 (50.1–79.5)
2 Weeks	9.77(±2.21)	37.2 (29.8–47.2)	87.02 (71.1–92.9)

GCF volume increased over 2 weeks (*P*<0.0001) and increases were significant at 1 and 2 weeks (*P*<0.019). The % of sites bleeding increased over 2 weeks (p<0.0001). The % of sites with visible plaque increased over 2 weeks (*P*< 0.0001) and increases were significant at 1 and 2 weeks (*P*<0.0001).

### Pyrosequencing summary

394 558 sequences with a mean length of 423 bases were obtained after initial quality filtering. Alignment to the SILVA reference database, subsequent screening of the alignment and removal of chimeras resulted in a final dataset of 344 267 high quality sequences (of which 31 604 were unique) with a mean length of 354 bases. This provided a final mean yield of 3742 (±789) sequences per sample for further analysis.

### OTU-based alpha and beta diversity of bacterial communities in plaque

**Figure 2:**
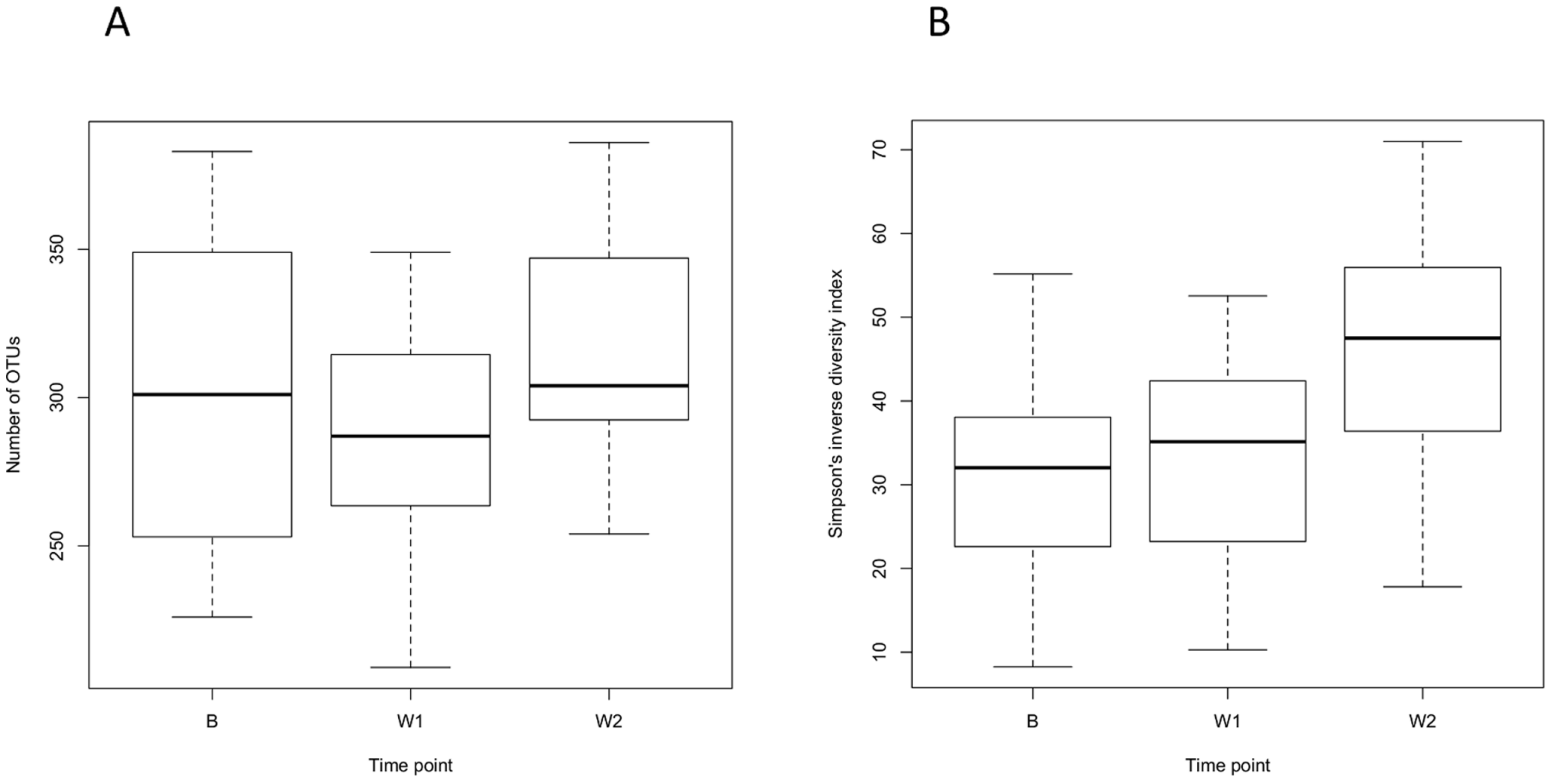
Richness and diversity of plaque during the induction of experimental gingivitis. Box-and-whisker plots comparing the species-level OTU richness and diversity of time points during experimental gingivitis. The top and bottom boundaries of the boxes show the 75^th^ and 25^th^ percentile and the ends of the whiskers show the maximum and minimum values. Bold lines within the boxes represent median values (50^th^ percentile). (**A**) Number of observed OTUs at baseline (B), one week (W1) and two weeks (W2). (**B**) Simpson's inverse diversity index at baseline (B), one week (W1) and two weeks (W2).

Clustering of sequences into OTUs at a distance of 0.015 resulted in 89 to 394 (median 299) species-level OTUs per plaque sample/bacterial community. Mean coverage of the communities according to Good's non-parametric was 96.9% (±0.7%). Chao1 estimates of total OTU richness ranged between 130 and 634 OTUs (median 464), whilst CatchAll gave estimates from 200 to 1690 (median 644). The observed OTU richness and diversity of communities (Simpson's inverse diversity index) at different sampling times of experimental gingivitis are shown in Figure 2. The richness of the communities across time points was significantly different (Friedman test, *P*<0.016). Using pairwise Wilcoxon signed-rank tests there was no significant difference between baseline communities and the one- and two-week communities. However, the number of OTUs was significantly higher in two-week communities compared to one-week communities (*P*<0.01). There was a significant difference in diversity across time points (Friedman test, *P*<0.00044). Pairwise Wilcoxon-signed rank tests showed that diversity was significantly higher in two-week communities compared to both baseline (*P*<0.0001) and one-week communities (*P*<0.0012), but that there was no significant difference between baseline and one-week communities. A table summarizing the alpha diversity parameters for each of the 92 samples analyzed is shown in the supporting information (Table S1). Only eight OTUs, assigned to the taxa: *Actinobaculum* sp. HOT183, *Campylobacter gracilis, Campylobacter showae, Cardiobacterium hominis, Fusobacterium nucleatum* subsp. *polymorphum, Lautropia mirabilis, Streptococcus mitis*/ HOT064/ HOT423/ HOTA95/ HOTE14, and *Veillonella parvula,* were detected in all healthy subjects' baseline samples. Three OTUs, assigned to the taxa *Fusobacterium nucleatum* subsp. *animalis, Fusobacterium nucleatum* subsp. *vincentii,* and *Streptococcus mitis*/ HOT064/ HOT423/ HOTA95/ HOTE14 were shared among all of the periodontitis patients.

Comparisons of the bacterial community membership (Jaccard index and unweighted UniFrac) of plaque samples using dendrograms, showed that communities from the different time points of experimental gingivitis clustered principally by subject (Figure S1). A separate comparison of baseline plaque communities from the healthy cohort to superficial plaque from chronic periodontitis patients showed some clustering by cohort (Figure S2). Interestingly, a separate analysis of superficial and subgingival plaque samples from periodontitis patients showed that communities clustered by patient rather than by the type of plaque sample (Figure S3). A parsimony analysis was conducted for each dendrogram to assess the significance of clustering patterns of groups and time points. There was a significant difference between the clustering of the baseline communities of healthy subjects and superficial plaque communities of periodontitis patients (*P*<0.036 and *P*<0.012 for Jaccard index and unweighted UniFrac dendrograms, respectively). There were no significant differences between the clustering of communities from different time points of experimental gingivitis or between superficial and subgingival plaque communities from periodontitis patients.

**Figure 3:**
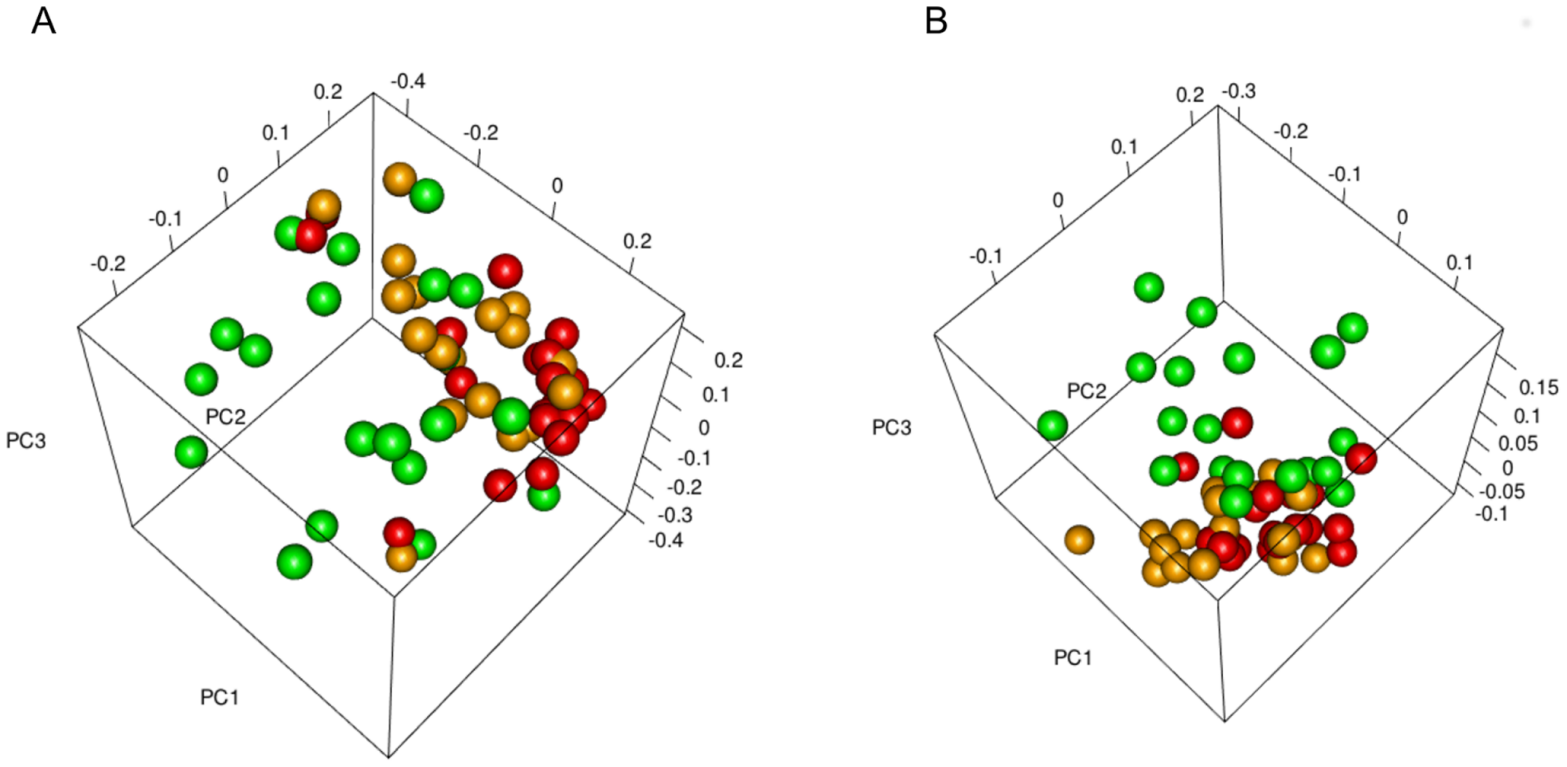
Shifts in bacterial community structure of plaque during the induction of experimental gingivitis. PCoA plots comparing community structure of plaque samples from different time points of experimental gingivitis. Baseline samples are colored green, one-week samples are orange and two- week samples are red. (**A**) PCoA based on the thetaYC calculator. PC1 = 12.11% of variance explained, PC2 = 8.89%, PC3 = 5.55% (**B**) PCoA based on the weighted UniFrac calculator. PC1 = 19.78% of variance explained, PC2 = 10.65%, PC3 = 5.65%.

**Figure 4:**
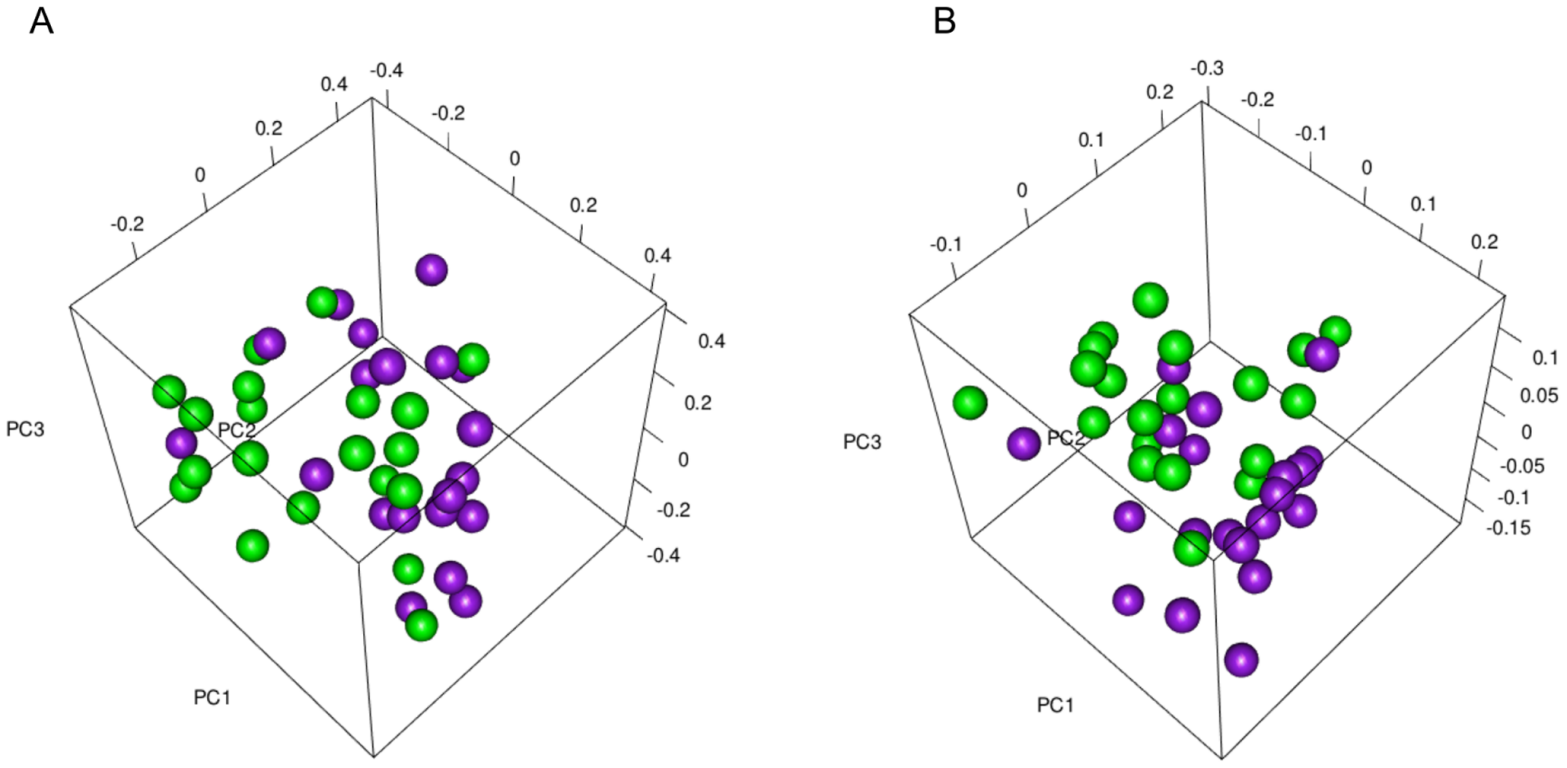
Bacterial community structure of plaque in health and chronic periodontitis. PCoA plots comparing community structure of baseline plaque samples from the healthy cohort to supragingival plaque samples of chronic periodontitis patients. Plaque communities from healthy individuals are colored green and those from periodontitis are colored purple. (**A**) PCoA based on the thetaYC calculator, PC1 = 16.38% of variance explained, PC2 = 10.09%, PC3 = 8.02% (**B**) PCoA based on the weighted UniFrac calculator, PC1 = 22.12% of variance explained, PC2 = 8.89%, PC3 = 6.85%.

Comparison of the bacterial community structure of plaque samples was performed using the thetaYC and weighted UniFrac metrics from which distance matrices were constructed and visualized using PCoA (Figures 3 and 4). For the experimental gingivitis cohort, both plots showed spatial separation of one- and two-week communities from baseline communities (Figure 3). In addition, separate PCoA analyses comparing superficial periodontitis communities with healthy baseline communities (Figure 4) and two-week gingivitis (not shown) communities showed separation in each case. AMOVA tests found an overall significant difference between the three time points of experimental gingivitis for both the thetaYC and weighted UniFrac distances (*P*<0.0016 and *P*<0.001, respectively). Pairwise AMOVA comparisons showed that there were significant differences between baseline and one-week communities (*P*<0.017 and *P*<0.001 for thetaYC and weighted UniFrac, respectively) and between baseline and two-week communities (*P*<0.0006 and *P*<0.001 for thetaYC and weighted UniFrac, respectively). However, there was no significant difference between one- and two-week communities (*P* = 0.526 and *P* = 0.098 for thetaYC and weighted UniFrac respectively). AMOVA tests also confirmed that there were significant differences between the baseline communities of healthy subjects and the superficial communities of the periodontitis patients (*P* = 0.0095 and *P*<0.001 for thetaYC and weighted UniFrac, respectively). Similarly, there was a significant difference between the two-week communities and the superficial periodontitis samples (*P*<0.0005 for both thetaYC and weighted UniFrac). There was no significant difference in the thetaYC or weighted UniFrac distances between the superficial and subgingival periodontitis samples from periodontitis patients by AMOVA testing.

### OTU-level composition and shifts of bacterial communities

**Figure 5:**
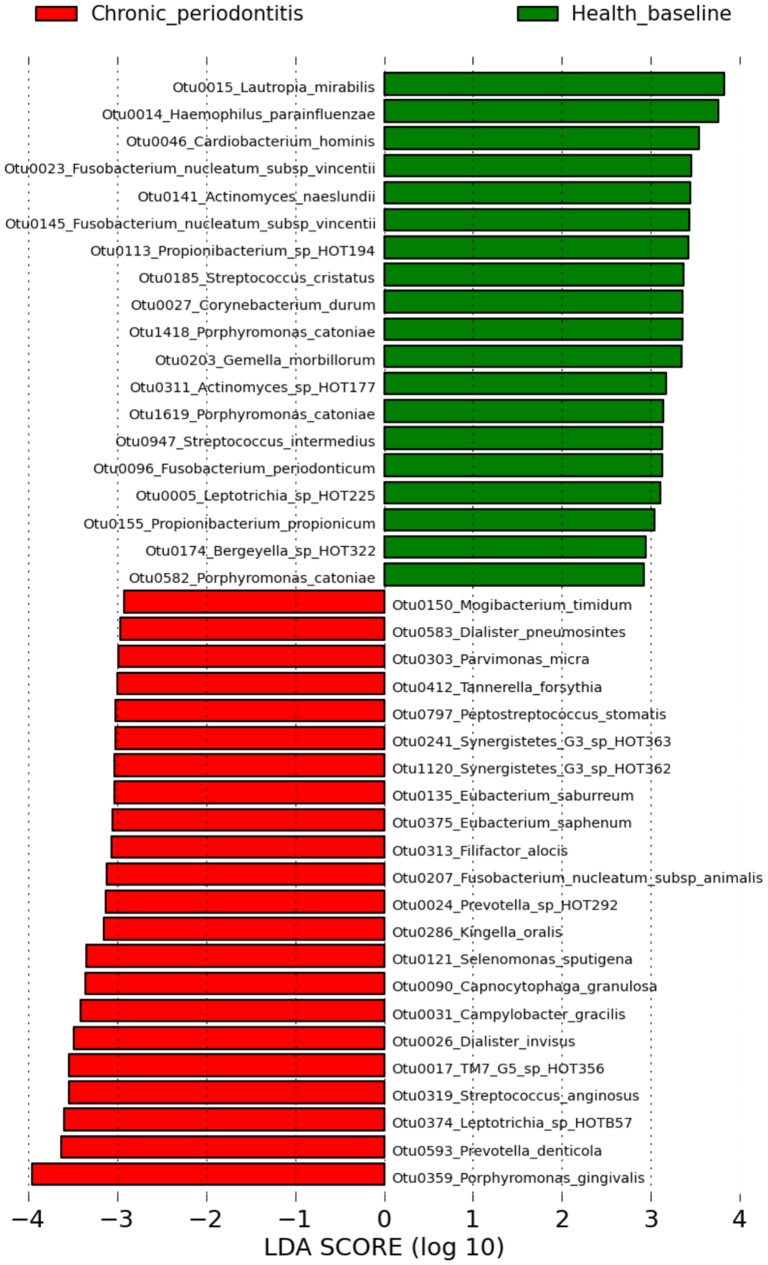
Detection of differentially abundant OTUs in health and chronic periodontitis. Differentially abundant OTUs between baseline plaque communities of the experimental gingivitis cohort and superficial plaque communities in chronic periodontitis patients as identified by LEfSe. OTUs are ranked by their LDA effect size. OTUs associated with healthy subjects are shown in green and OTUs associated with chronic periodontitis are shown in red.

The 10 OTUs detected with the highest relative abundance across all 92 samples were assigned to the taxa *Streptococcus sanguinis, Rothia dentocariosa, Veillonella parvula, Fusobacterium nucleatum* subsp. *polymorphum, Streptococcus mitis*/ HOT064/ HOT423/ HOTA95/ HOTE14, *Streptococcus cristatus*/ HOT071, *Fusobacterium nucleatum* subsp. *vincentii, Lautropia mirabilis, Porphyromonas gingivalis* and *Leptotrichia buccalis.* The intra-sample relative abundances of the top 50 OTUs detected are shown in the supporting information (Dataset S1). Statistical analysis using linear models implemented in MaAsLin was initially performed to determine correlations of OTUs with sampling times during experimental gingivitis. A full list of the resulting OTUs that were positively or negatively correlated with one- and/or two-week time points of experimental gingivitis are shown in Table S2. Box plots showing changes in relative abundance over time for the most significant OTUs are shown in Figure S4. A second analysis in MaAsLin included bleeding on probing (BoP) scores and the baseline and two-week time points. OTUs were statistically associated with the clinical condition indicated by BoP scores, rather than the time point at which the samples were collected. The OTUs identified as *Lautropia* sp. HOTA94, *Lachnospiraceae* sp. HOT100, *Prevotella oulorum* and *Fusobacterium nucleatum* subsp. *polymorphum* were most significantly positively correlated with BoP (*P* and *Q* values <0.05) whilst an OTU identified as *Rothia dentocariosa* was most significantly negatively correlated with BoP. A full list of OTUs that were correlated with BoP scores is shown in Table S3. Linear discriminant analysis using LEfSe was used to detect OTUs that had significantly different relative abundances between chronic periodontitis (superficial plaque) and health (baseline plaque). A total of 41 OTUs were found to be significantly differentially abundant between these groups (Figure 5). An OTU identified as *Porphyromonas gingivalis* was most strongly associated with chronic periodontitis.

### BLAST identification using the HOMD reference set

#### BLAST summary

Following BLAST, 331 398 of the 344 267 sequences (96.3%) were mapped to taxa in the HOMD reference set. The 331 398 sequences were assigned to a total of 11 phyla, 126 genera and 567 species-level phylotypes/groups. 3.7% of the 344 267 sequences were not assigned (<98.5%) to any known phylotypes in the HOMD reference set.

#### Phylum level composition

The predominant phyla detected across all plaque samples in order of mean relative abundance in samples were *Firmicutes* (31.3%), *Fusobacteria* (19.0%), *Bacteroidetes* (18.9%), *Actinobacteria* (14.0%), and *Proteobacteria* (13.9%). Other phyla detected, but which were not present in every sample, included TM7, *Synergistetes*, *Spirochaetes*, SR1, *Chloroflexi* and GN02. There was considerable inter-individual variability in the relative abundance of phyla, both among healthy individuals and patients with chronic periodontitis. However, comparison of the mean relative abundances of the predominant phyla for each cohort (Figure S5) showed that *Proteobacteria* were significantly more abundant in health (two sample *t*-test: *P*<0.0056) whilst *Bacteroidetes* were significantly more abundant in periodontitis (*P*<0.0039). Furthermore, the phyla *Synergistetes* and *Spirochaetes* were detected in 90% and 95% of the periodontitis patients and 40% and 80% of the healthy subjects, respectively. The phylum *Chloroflexi* was not detected in health but found in 15% of the periodontitis patients at low levels. Phylum-level shifts within and across all subjects were observed during experimental gingivitis (Figure S6). Specifically, the relative abundance of *Actinobacteria* was significantly higher at baseline compared to one and two weeks (paired *t*-test: *P<*0.001 for both comparisons), whilst the *Bacteroidetes* were significantly higher in one- and two-week samples compared to baseline (*P*<0.001 for both comparisons). The shifts in phyla during experimental gingivitis, however, showed considerable variability among individuals. For example, subject four showed a striking drop in their relative abundance of *Actinobacteria*, from 41.6% at baseline to 9.3% after two weeks. In contrast, the relative abundances of *Actinobacteria* in subject five were 15.1% at baseline and 18.5% after two weeks.

### Culture-based community analysis

Baseline and two week plaque samples were cultured for 11 of the healthy subjects. For one subject, only the baseline sample was obtained (subject two). 1935 of 1956 isolates were assigned to taxa in the HOMD extended reference set V1.1 using BLAST (≥98.5% sequence identity). These sequences represented five phyla (*Actinobacteria*, *Bacteroidetes*, *Firmicutes*, *Fusobacteria*, and *Proteobacteria*) and 43 genera. The phylotypes/ groups detected with the highest relative abundance using both incubations (anaerobic and air +5% C0_2_) were *Streptococcus sanguinis* and the *Actinomyces naeslundii* group. *S. sanguinis* was the most abundant isolate at baseline with a median relative abundance of 18.8% and 13.8% using air +5% C0_2_ and anaerobic incubations respectively. After two weeks the *A. naeslundii* group was the most abundant (21.7% and 14.0% using air +5% C0_2_ and anaerobic incubations respectively). The observed OTU richness and diversity of baseline and two-week communities by culture is summarized in Table 2.

**Table 2:** Alpha diversity of plaque samples as analyzed by culture.

Time point (incubation)	No. of observed OTUs	Simpson's inverse diversity index	Chao 1 total OTU richness estimate
	Median (IQR)	Median (IQR)	Median (IQR)
Baseline (air +5% C0_2_)	14.5 (11.0–21.0)	10.9 (6.6–17.9)	19.1 (15.4–36.0)
2 Weeks (air +5% C0_2_)	18.5 (15.3–22.0)	14.6 (9.3–19.5)	30.4 (23.2–43.2)
Baseline (Anaerobic)	22.5 (18.3–24.8)	16.8 (12.7–29.2)	51.3 (31.0–71.2)
2 Weeks (Anaerobic)	24.5 (21.5–28.3)	25.0 (15.8–34.6)	57.9 (38.0–70.4)

A number of taxa isolated in culture were not detected by pyrosequencing among the same samples, including *Actinomyces* sp. HOT172, *Capnocytophaga* sp. HOT380, *Staphylococcus epidermidis* group, *Staphylococcus hominis*, *Staphylococcus warneri, Paenibacillus* HOTA06, and *Propionibacterium acnes*/HOT193. No representatives of the phyla GN02, *Spirochaetes*, SR1, *Synergistetes*, or TM7, were detected by culture.

### Novel oral taxa

12 869 sequences in the pyrosequencing dataset were not mapped to any taxa in HOMD (<98.5% sequence similarity). These sequences represented lineages within a number of different phyla and many were found in multiple samples in both the experimental gingivitis and chronic periodontitis cohorts. Seven of these phylotypes/groups were investigated further. Virtually full length sequences were obtained for all of these phylotypes/groups using specific 16 S rRNA primers for targeted PCR, as well as for two novel cultured isolates and made available on GenBank and submitted to HOMD. A summary of these taxa, their closest known phylogenetic relatives, and Genbank accession numbers are shown in Table 3. Of particular interest was a deep branching lineage within the class *Mollicutes* representing a new order. The prevalence of these taxa in the experimental gingivitis and chronic periodontitis cohorts is shown in Table S4 and phylogenetic trees for each taxon are shown in Figures S7–15.

**Table 3:** Oral taxa targeted for full-length 16S rRNA gene sequencing.

Closest phylogenetic relative in HOMD (% sequence identity)	Name and accession no. of closest match in Genbank (% sequence identity)	Clone/ Isolate	Genbank accession no.	HOMD oral taxon no.
*Bacillus* sp. HOT A03 (84%)	Uncultured bacterium clone 4_11, HE681226 (99%)	Clone	KC203059	HOT 906
*Microlunatus* sp. HOT C95 (93.5%)	Uncultured *Propionibacteriaceae* bacterium clone 08_3_G04, GU227180 (99%)	Clone	KC203064	HOT 915
*Prevotella* sp. HOT 473 (94.3%)	Uncultured *Prevotellaceae* bacterium clone 601F05, AM420222 (99%)	Clone	KC203063	HOT 914
*Actinomyces* sp. HOT 449 (94.8%)	Uncultured bacterium clone 070050_018, JQ466816 (99%)	Clone	KC203057	HOT 897
*Bergeyella* sp. HOT 322 (96.3%)	Uncultured bacterium clone rRNA004, AY958777 (99%)	Clone	KC203058	HOT 900
*Tannerella* sp. HOT 808 (97.9%)	Uncultured *Tannerella* sp. clone 402C09, AM420141 (99%)	Clone	KC203065	HOT 916
*Leptotrichia hofstadii* (97.9%)	Uncultured *Leptotrichia* sp. clone 303F08, AM420110 (99%)	Clone	KC203062	HOT 909
*Aggregatibacter* sp. HOT 513 (97.9%)	Uncultured bacterium clone P1D1-738, EF511870 (98%)	Isolate	KC203060	HOT 898
*Capnocytophaga* sp. HOT 336 (98.3%)	*Capnocytophaga* sp. P2 oral strain P4_P12, AY429469 (98%)	Isolate	KC203061	HOT 903

## Discussion

This is the first study to use 454-pyrosequencing to examine the bacterial composition of dental plaque in experimental gingivitis, and one of few reported longitudinal investigations of the oral microbiome. The results of this study have shown that, in the absence of oral hygiene, the transition from periodontal health to gingivitis is accompanied by a shift in the bacterial community structure of plaque and an increase in bacterial community diversity. In addition, the results demonstrated significant differences in both the membership and structure of analogous health- and chronic periodontitis-associated plaque samples, and confirmed the association of particular species previously associated with chronic periodontitis [18], [19].

A number of previous high-throughput 16S rRNA sequencing studies characterized oral bacterial communities to the phylum or genus level only [49]–[51]. It is important to distinguish taxa at the species-level, as different species within the same phylum and/or genus may be health-associated or pathogenic/disease-associated. The targeting of a highly variable region of the 16S rRNA gene (V1–V3) and the use of a curated human oral 16S rRNA gene reference set (HOMD), enabled the identification of OTUs (clustered at a distance of 0.015) to species-level where possible. Whilst some studies [18]–[20] have also recently reported species-level 16S rRNA gene pyrosequencing analysis of the bacterial communities in periodontal health, gingivitis and chronic periodontitis, these studies were cross-sectional in nature and did not examine changes in the same individuals during the transition from health to disease. In the present study a highly species-rich bacterial community (201–383 OTUs per sample) was revealed in early health-associated plaque. This richness is considerably higher than indicated by the culture data of this study (Table 2) and in previous studies characterizing the oral microbiome in health. Aas et al. [13] found between 12 and 27 species-level phylotypes on tooth surfaces and between four and 21 in subgingival plaque, while Bik et al. [15] detected between 65 and 128 species level OTUs in pooled samples from different oral surfaces. The number of species-level OTUs per plaque sample observed in health in the present study is in a similar range to other recent 16S rRNA pyrosequencing studies. Zaura et al. [52] found on average 266 species-level phylotypes (97% sequence similarity) per sample and Griffen et al. [18] detected between 100 and 300 phylotypes (98% sequence similarity) per individual. However, Huang et al. [20] reported the presence of 379–684 species-level OTUs (97% sequence similarity) in the supra-gingival plaque of healthy individuals. Comparison of the numbers of observed OTUs, or phylotypes, between this and other studies, though, is complicated by differing methodologies such as, but not limited to, the use of different regions of the 16S rRNA gene and different sequence similarity cut-offs for OTU clustering or BLAST classification. A recent study [22] analyzed a mock bacterial community using pyrosequencing and showed that despite de-noising and stringent quality filtering, additional erroneous OTUs were detected, indicating that pyrosequencing may still over-estimate the number of OTUs present.

The species-level OTU richness detected by pyrosequencing was not significantly higher in subjects after one and two weeks without oral hygiene. This was perhaps surprising given findings to the contrary in a previous culture-based experimental gingivitis study [9]. These observations are, however, similar to that of Huang et al. [20] who, using pyrosequencing, did not note any significant difference in richness between plaque samples from healthy individuals and those with gingivitis. The significant increase in community diversity (Simpson's inverse diversity index), after two weeks of experimental gingivitis, in the absence of significantly increased richness, indicated that the increased diversity was mainly a result of increasing evenness. This suggests that as plaque accumulated, species that dominated the early communities in health decreased in relative abundance over time whilst previously minor constituent species increased in relative abundance, resulting in a more even distribution of species after two weeks of plaque accumulation. Significant differences between the bacterial community structures (using both OTU- and phylogenetic-based analyses) of plaque in health (baseline) and gingivitis (one- and two-week plaque samples) were shown by the pyrosequencing data. However, clustering comparisons on the basis of community membership indicated that inter-individual differences were greater. This was supported by the low number of OTUs that were shared between all 20 healthy subjects, suggesting a small core/shared oral microbiota in plaque at this taxonomic level.

The PCoA plots revealed a shift in bacterial community structure as gingivitis developed in the subjects following the withdrawal of oral hygiene. It is interesting to note, however, that the PCoA plots also indicated considerable variability in community structure among the healthy subjects. This variability was evident both at the phylum- and species-level. For example, the relative abundance of *Actinobacteria* ranged between 8.4% and 55.9% in baseline samples. At the species-level, OTUs that were dominant in some individuals' baseline samples were detected only at low relative abundances, or not at all, in others. An OTU identified as *Neisseria flavescens*/*subflava* was the dominant OTU at baseline in subject 16 (8.81% of the sequences) but was not detected in 11 of the healthy subjects. It would be useful if future studies examining the microbial composition of plaque in health and periodontal disease include a greater number of subjects, as the relatively low number (20 healthy subjects and 20 chronic periodontitis patients) in the present study was a limitation given the observed inter-individual variability. Continuing advances in high-throughput sequencing technology may facilitate this. Despite this inter-individual variability, the present study identified a number of OTUs that showed significant changes in relative abundance after one and two weeks of experimental gingivitis. The analyses also identified OTUs that were negatively or positively correlated with bleeding on probing (BoP) scores. OTUs that decreased in relative abundance over time and that were negatively correlated with BoP, were predominantly aerobic and facultatively anaerobic Gram-positive cocci and rods, including members of the genera *Actinomyces*, *Rothia*, and *Streptococcus*. It has been previously shown that *Streptococcus* spp., *Actinomyces* spp., and *Rothia* spp. are among the earliest colonizers of the tooth surface [21], [53] and are prevalent in the mouths of healthy individuals [13], [15] so it was unsurprising that members of these genera were abundant in health. An OTU identified as *Rothia dentocariosa* showed the most significant negative correlation with one- and two-week time points of experimental gingivitis, and with increased BoP scores. *R. dentocariosa* has previously been identified as a common constituent of the oral microbiome in health, particularly on tooth surfaces [13], [15], [50] and the genus *Rothia* has been associated with oral health. [18], [54]. Furthermore, a recent pyrosequencing study found that *R. dentocariosa* dominated the health-associated subgingival plaque communities analyzed [19]. The OTUs that increased in relative abundance as gingivitis developed and that were positively correlated with BoP scores were mostly Gram-negative taxa of the genera *Campylobacter*, *Fusobacterium*, *Lautropia, Leptotrichia, Porphyromonas, Selenomonas,* and *Tannerella.* Among those that have been previously cultivated, many were obligate anaerobes. These findings are largely in accordance with the observations of Theilade et al. [7], who, in an early experimental gingivitis study, reported an increase in the proportion of Gram-negative cocci and rods as well as filaments, spirilla and spirochetes as gingivitis developed. The OTUs most strongly positively correlated with increased BoP scores included the unnamed phylotypes *Lachnospiraceae* [G-2] sp. HOT100 and *Lautropia* sp. HOTA94, as well as the named previously cultured organisms *Fusobacterium nucleatum* subsp. *polymorphum* and *Prevotella oulorum.* An interesting observation of this study was the increased relative abundance in one- and two-week time points, and positive correlation with BoP, of an OTU identified as *Tannerella* sp. HOT286 (also known as oral clone BU063). This phylotype, a close relative of the putative periodontal pathogen *Tannerella forsythia*, was previously associated with periodontal health in a study using PCR to compare its prevalence in the 25% of a population with the most severe periodontitis to its prevalence in the 25% of the population with the best periodontal health [55]. In that study, the relatively healthy subjects included some with pocket depths and attachment loss up to a maximum of 5 mm and, unlike the present study, the extent of gingival inflammation was not reported. [55]. Interestingly, *Tannerella* sp. oral clone BU063 was found to be more prevalent among individuals with gingivitis and necrotizing ulcerative gingivitis than in those with periodontitis in another study using fluorescent *in situ* hybridization [56]. Furthermore, Huang et al. [20] recently reported a significantly higher relative abundance of *Tannerella* sp. BU063 in the plaque of individuals with gingivitis than in healthy individuals.

The majority of OTUs that had a significantly higher relative abundance in chronic periodontitis patients than in healthy subjects were taxa that have been previously associated with periodontitis. However, one strongly associated OTU (*Leptotrichia* sp. HOTB57) may represent an additional taxon to add to this expanding list. *Porphyromonas gingivalis* had the greatest effect size among the associated OTUs and has been previously associated with periodontitis on the basis of both culture-dependent and independent studies [14], [16], [57]. Interestingly, *Prevotella denticola* was among the other strongly periodontitis-associated OTUs. *P. denticola* was previously found to increase in incidence with increasing severity of periodontal disease based on culture [58], was detected at higher prevalence in periodontitis patients than healthy subjects using species-specific PCR [16] and was strongly associated with chronic periodontitis in another recent pyrosequencing study [18]

The volunteer group of healthy subjects were all clinical staff within the Dental Institute who were highly motivated and well informed in the practise of effective oral hygiene. The clinical condition in severe chronic periodontitis contributed a substantially different clinical environment for comparison, but a convenience sample of the 20 patients with severe chronic periodontitis can not be said to represent the entire population with periodontitis. The patients with periodontitis were significantly older than the healthy volunteers and this is a limitation of the current study. For ethical reasons it would not be appropriate to monitor changes in the microbiota whilst allowing irreversible destructive disease to progress over a number of years without intervention. The effects of patient age on the microbiota can not be easily separated from the effect of the different microbial habitat which develops as the patient ages and disease progresses. This study could not have been ethically designed to monitor the microbiota as gingivitis progressed to periodontitis or as periodontitis increased in severity with age. More extensive studies would be required to compare different types of periodontal disease, different levels of disease in different age groups and populations before a truly comprehensive description of the periodontal microbiome could be described with complete confidence. However, within these limitations, the current work has successfully applied deep sequencing technology to monitor short term changes in the microbiota during the induction of reversible mild periodontal disease and contrasted it with the microbiota of a group of patients with severe and irreversible periodontal disease.

In conclusion, this study has shown the presence of a highly rich bacterial biota in health-associated plaque and determined longitudinal shifts in bacterial community structure as plaque accumulates and gingivitis develops. The analyses both identified new health- and gingivitis-associated taxa and confirmed the association of a number of putative periodontal pathogens with chronic periodontitis. Further investigation of these taxa may lead to the development of novel therapies aiming to prevent the early stages of periodontal disease.

## Supporting Information

Figure S1 **Clustering of plaque communities in experimental gingivitis.** Dendrogram of plaque samples from all time points of experimental gingivitis compared based on their community membership using the Jaccard index. HS = healthy subject, B = baseline, 1W = one week, 2W = two weeks. Numbers indicate subject number.
(PDF)

Figure S2 **Clustering of plaque communities in health and chronic periodontitis.** Dendrogram of plaque samples from the baseline time point of the experimental gingivitis cohort and superficial plaque samples from patients with periodontitis, compared based on their community membership using the Jaccard index. HS = healthy subject, B = baseline, CP = chronic periodontitis patients. Numbers indicate subject or patient number.
(PDF)

Figure S3 **Clustering of superficial and subgingival plaque communities in chronic periodontitis.** Dendrogram of superficial and subgingival plaque samples from patients with chronic periodontitis, compared based on their community membership using the Jaccard index. CP = chronic periodontitis patients, Sub = subgingival plaque. Numbers indicate patient number.
(PDF)

Figure S4 **Changes in relative abundance of OTUs during the induction of experimental gingivitis.** Box plots showing significant changes in relative abundance of OTUs during induction of experimental gingivitis. Time 1  =  baseline, Time 2  = 1 week, Time 3  = 2 weeks. All OTUs shown are *P*<0.05, *Q*<0.05. Correlation coefficients are shown in parentheses.
(TIF)

Figure S5 **Relative abundances of the predominant phyla in health and chronic periodontitis.** Histogram comparing the mean relative abundances of the predominant phyla detected in healthy subjects (baseline) and chronic periodontitis patients (superficial plaque). Statistically significant differences as indicated by two-sample t-tests are highlighted with an * and error bars shown are the standard error of the mean (SEM).
(TIF)

Figure S6 **Relative abundances of the predominant phyla during the induction of experimental gingivitis.** Histogram chart comparing the mean relative abundances of the predominant phyla at the different time points of experimental gingivitis. Statistically significant differences as indicated by two-sample t-tests are highlighted with an * and error bars shown are the standard error of the mean (SEM).
(TIF)

Figure S7 Phylogenetic tree based on 16S rRNA gene comparisons showing the relationship between *Mollicutes*_CP6_C1, members of the *Firmicutes* phylum and other phyla found in the oral cavity. The tree was constructed using the neighbor-joining method from a distance matrix constructed from aligned sequences using the Jukes-Cantor correction. Numbers represent bootstrap values for each branch based on data from 500 trees. Scale bars show the number of nucleotide substitutions per site.
(PDF)

Figure S8 Phylogenetic tree based on 16S rRNA gene comparisons showing the relationship between *Propionibacteriaceae* HS10_B_C3 and members of the phylum *Actinobacteria*. The tree was constructed using the neighbor-joining method from a distance matrix constructed from aligned sequences using the Jukes-Cantor correction. Numbers represent bootstrap values for each branch based on data from 500 trees. Scale bars show the number of nucleotide substitutions per site.
(PDF)

Figure S9 Phylogenetic tree based on 16S rRNA gene comparisons showing the relationship between, *Alloprevotella*_HS3_1W_C6 and members of the genera *Alloprevotella* and *Prevotella*. The tree was constructed using the neighbor-joining method from a distance matrix constructed from aligned sequences using the Jukes-Cantor correction. Numbers represent bootstrap values for each branch based on data from 500 trees. Scale bars show the number of nucleotide substitutions per site.
(PDF)

Figure S10 Phylogenetic tree based on 16S rRNA gene comparisons showing the relationship between, *Actinomyces*_HS14_2W_C6 and members of the genus *Actinomyces*. The tree was constructed using the neighbor-joining method from a distance matrix constructed from aligned sequences using the Jukes-Cantor correction. Numbers represent bootstrap values for each branch based on data from 500 trees. Scale bars show the number of nucleotide substitutions per site.
(PDF)

Figure S11 Phylogenetic tree based on 16S rRNA gene comparisons showing the relationship between *Bergeyella*_HS1_1W_C3, members of the genus *Bergeyella* and other members of the class *Flavobacteria* in the phylum *Bacteroidetetes*. The tree was constructed using the neighbor-joining method from a distance matrix constructed from aligned sequences using the Jukes-Cantor correction. Numbers represent bootstrap values for each branch based on data from 500 trees. Scale bars show the number of nucleotide substitutions per site.
(PDF)

Figure S12 Phylogenetic tree based on 16S rRNA gene comparisons showing the relationship between *Tannerella* CP6_C2 and members of the genera *Tannerella* and *Porphyromonas*. The tree was constructed using the neighbor-joining method from a distance matrix constructed from aligned sequences using the Jukes-Cantor correction. Numbers represent bootstrap values for each branch based on data from 500 trees. Scale bars show the number of nucleotide substitutions per site.
(PDF)

Figure S13 Phylogenetic tree based on 16S rRNA gene comparisons showing the relationship between *Leptotrichia* CP4_C6 and members of the genus *Leptotrichia*. The tree was constructed using the neighbor-joining method from a distance matrix constructed from aligned sequences using the Jukes-Cantor correction. Numbers represent bootstrap values for each branch based on data from 500 trees. Scale bars show the number of nucleotide substitutions per site.
(PDF)

Figure S14 Phylogenetic tree based on 16S rRNA gene comparisons showing the relationship between *Aggregatibacter*_HS19_2W_I12 and members of the genera *Aggregatibacter* and *Haemophilus*. The tree was constructed using the neighbor-joining method from a distance matrix constructed from aligned sequences using the Jukes-Cantor correction. Numbers represent bootstrap values for each branch based on data from 500 trees. Scale bars show the number of nucleotide substitutions per site.
(PDF)

Figure S15 Phylogenetic tree based on 16S rRNA gene comparisons showing the relationship between *Capnocytophaga*_HS5_2W_I24 and members of the genus *Capnocytophaga*. The tree was constructed using the neighbor-joining method from a distance matrix constructed from aligned sequences using the Jukes-Cantor correction. Numbers represent bootstrap values for each branch based on data from 500 trees. Scale bars show the number of nucleotide substitutions per site.
(PDF)

Table S1 **Alpha diversity of plaque samples.**
(DOC)

Table S2 **OTUs associated with time points of experimental gingivitis.** OTUs were associated with time points of experimental gingivitis using Multivariate Association with Linear Models (MaAsLin). OTUs are ranked according to their *P* value. OTUs listed have *P* values <0.05.
(DOC)

Table S3 **OTUs associated with bleeding on probing scores.** OTUs were associated with BoP using Multivariate Association with Linear Models (MaAsLin). OTUs are ranked according to their *P* value. OTUs listed have *P* values <0.05.
(DOC)

Table S4 **Prevalence of novel taxa in the experimental gingivitis and chronic periodontitis cohorts.**
(DOC)

Dataset S1 **Relative abundances of the top 50 OTUs in plaque samples.** HS  =  Healthy subject, 1W  = 1 week, 2W  = 2 weeks, CP  =  Chronic periodontitis patient, Sub  =  subgingival plaque, Numbers indicate subject or patient number. Values given are percentage of total sequences.
(XLS)
