# Supplementary figures and images for: Bacterial Community Development in Experimental Gingivitis
Source: PLoS One. 2013 Aug 14;8(8):e71227. doi: 10.1371/journal.pone.0071227 (PMC3743832; doi:10.1371/journal.pone.0071227)

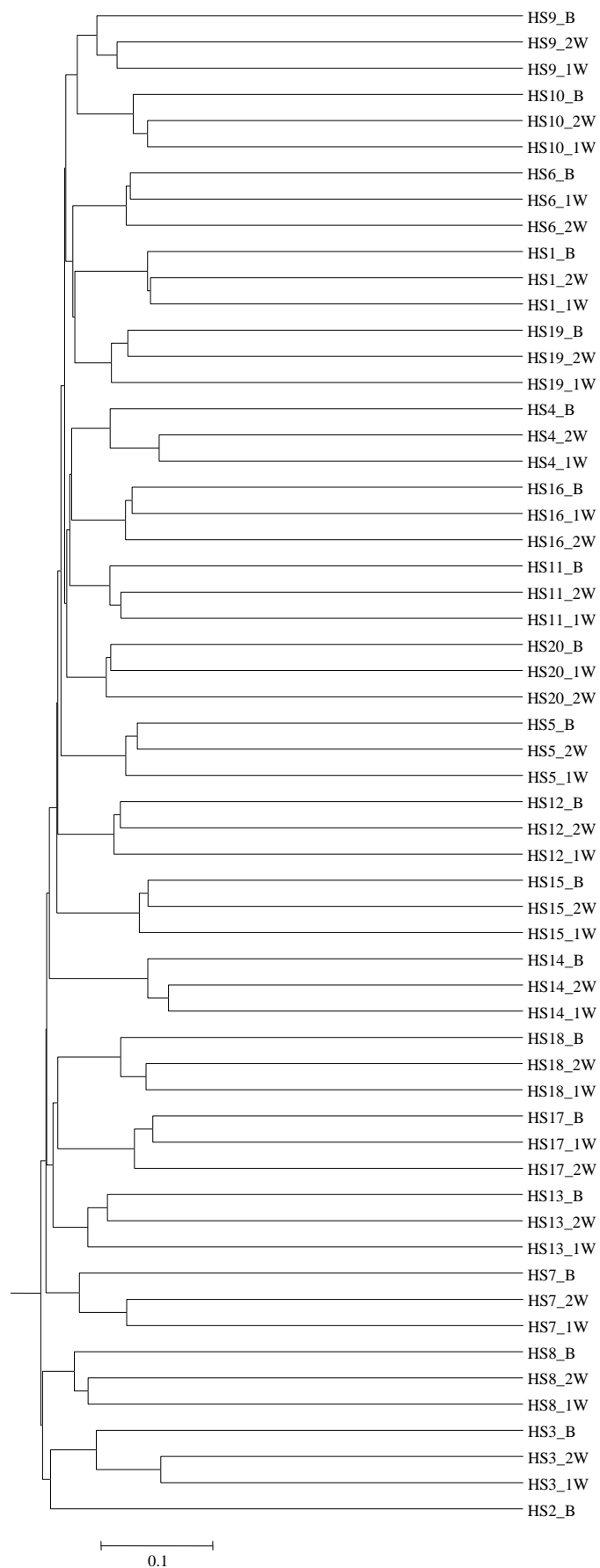

Supplement: Figure S1 — Clustering of plaque communities in experimental gingivitis. Dendrogram of plaque samples from all time points of experimental gingivitis compared based on their community membership using the Jaccard index. HS = healthy subject, B = baseline, 1W = one week, 2W = two weeks. Numbers indicate subject number. (PDF) [file pone.0071227.s001.pdf]

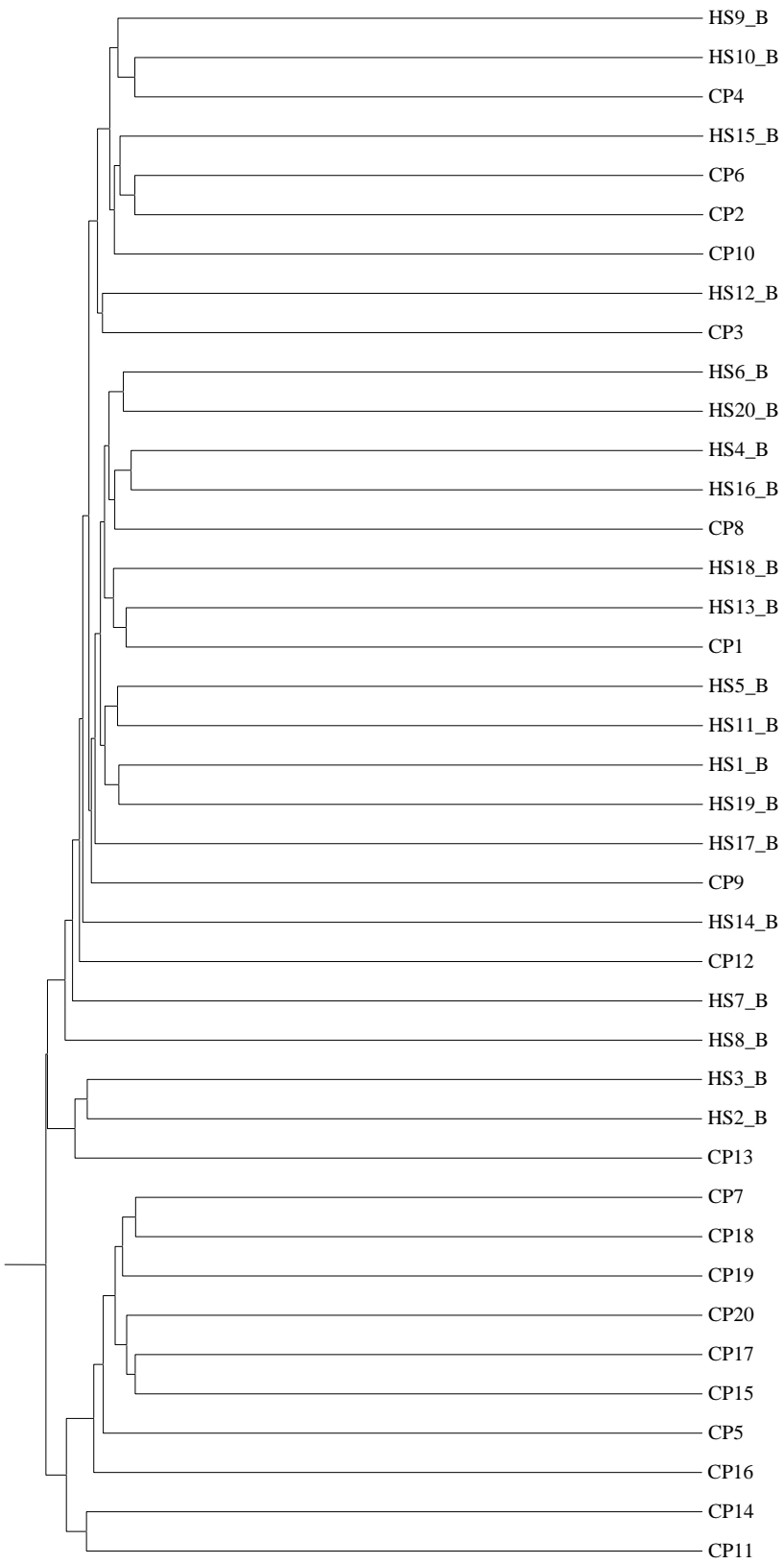

Supplement: Figure S2 — Clustering of plaque communities in health and chronic periodontitis. Dendrogram of plaque samples from the baseline time point of the experimental gingivitis cohort and superficial plaque samples from patients with periodontitis, compared based on their community membership using the Jaccard index. HS = healthy subject, B = baseline, CP = chronic periodontitis patients. Numbers indicate subject or patient number. (PDF) [file pone.0071227.s002.pdf]

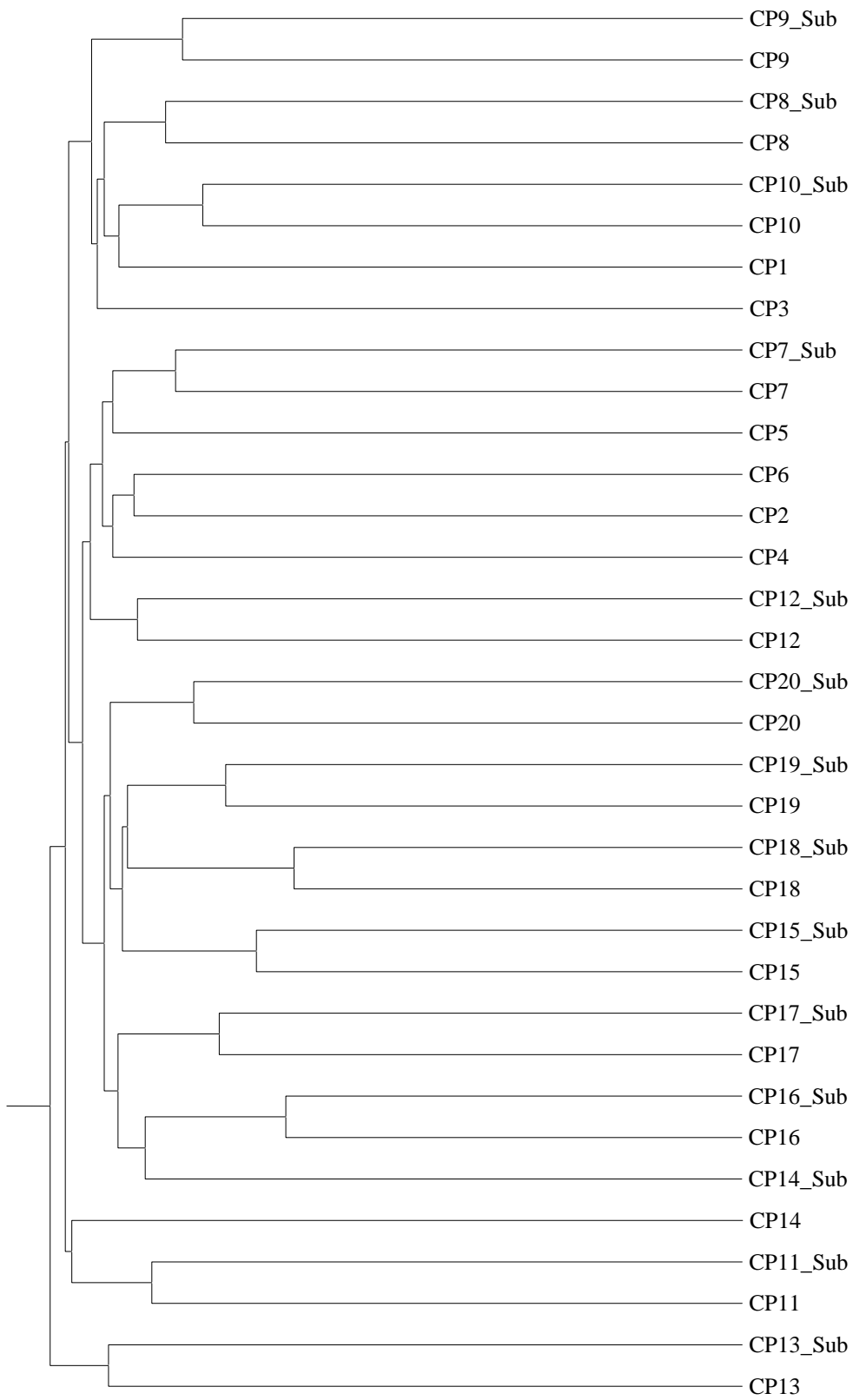

0.1

Supplement: Figure S3 — Clustering of superficial and subgingival plaque communities in chronic periodontitis. Dendrogram of superficial and subgingival plaque samples from patients with chronic periodontitis, compared based on their community membership using the Jaccard index. CP = chronic periodontitis patients, Sub = subgingival plaque. Numbers indicate patient number. (PDF) [file pone.0071227.s003.pdf]

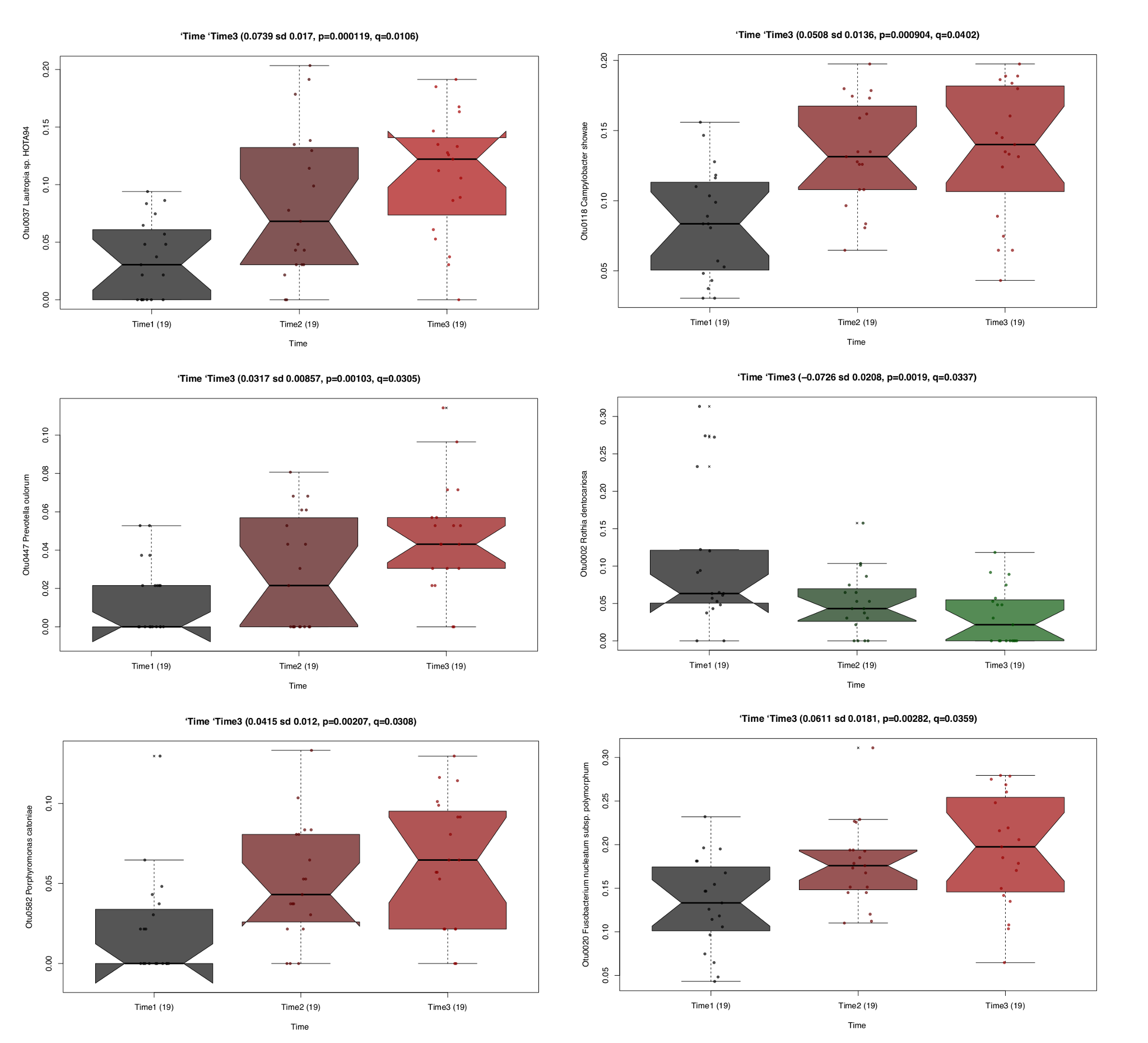

Supplement: Figure S4 — Changes in relative abundance of OTUs during the induction of experimental gingivitis. Box plots showing significant changes in relative abundance of OTUs during induction of experimental gingivitis. Time 1 = baseline, Time 2 = 1 week, Time 3 = 2 weeks. All OTUs shown are P<0.05, Q<0.05. Correlation coefficients are shown in parentheses. (TIF) [file pone.0071227.s004.tif]

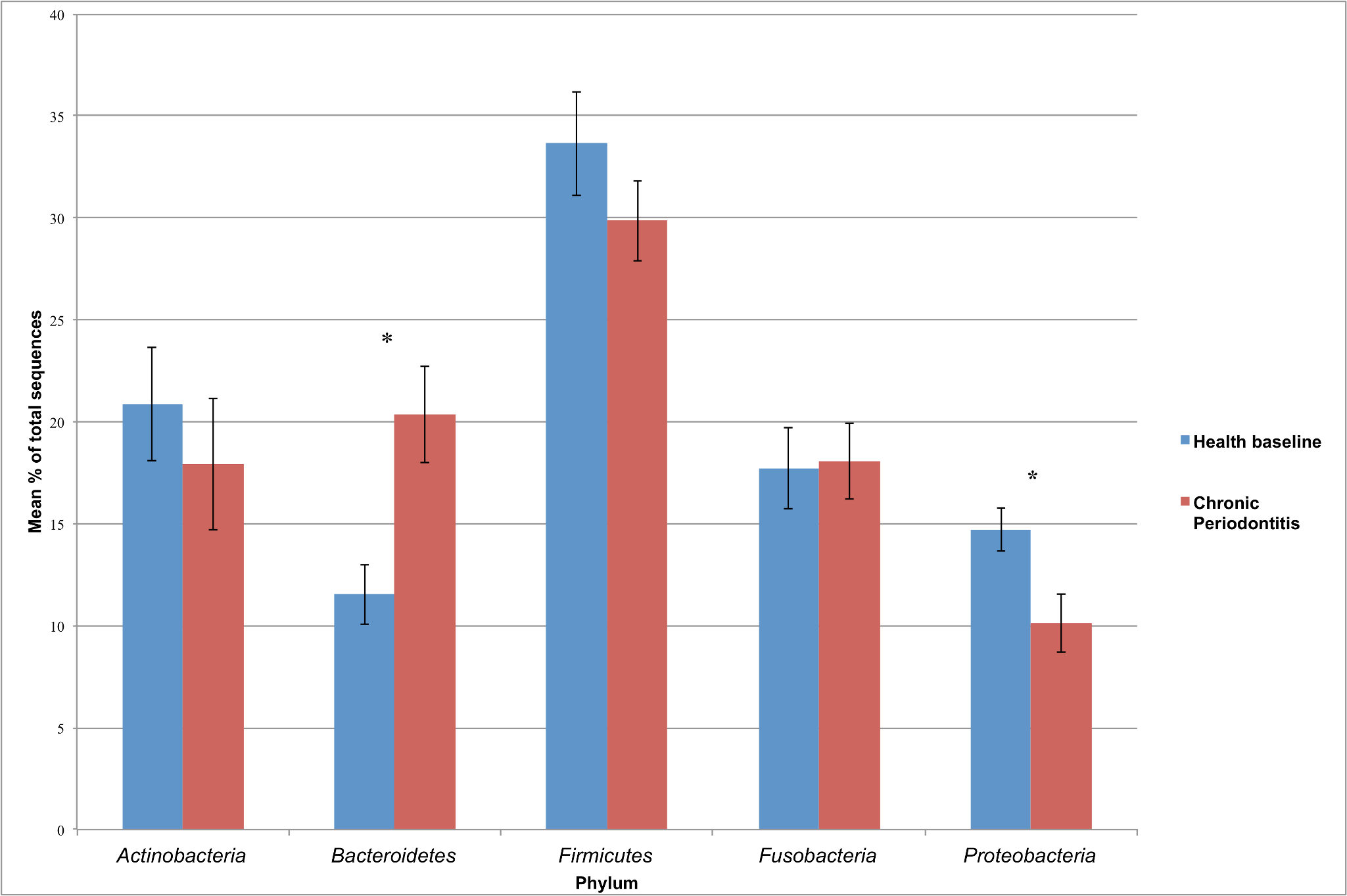

Supplement: Figure S5 — Relative abundances of the predominant phyla in health and chronic periodontitis. Histogram comparing the mean relative abundances of the predominant phyla detected in healthy subjects (baseline) and chronic periodontitis patients (superficial plaque). Statistically significant differences as indicated by two-sample t-tests are highlighted with an * and error bars shown are the standard error of the mean (SEM). (TIF) [file pone.0071227.s005.tif]

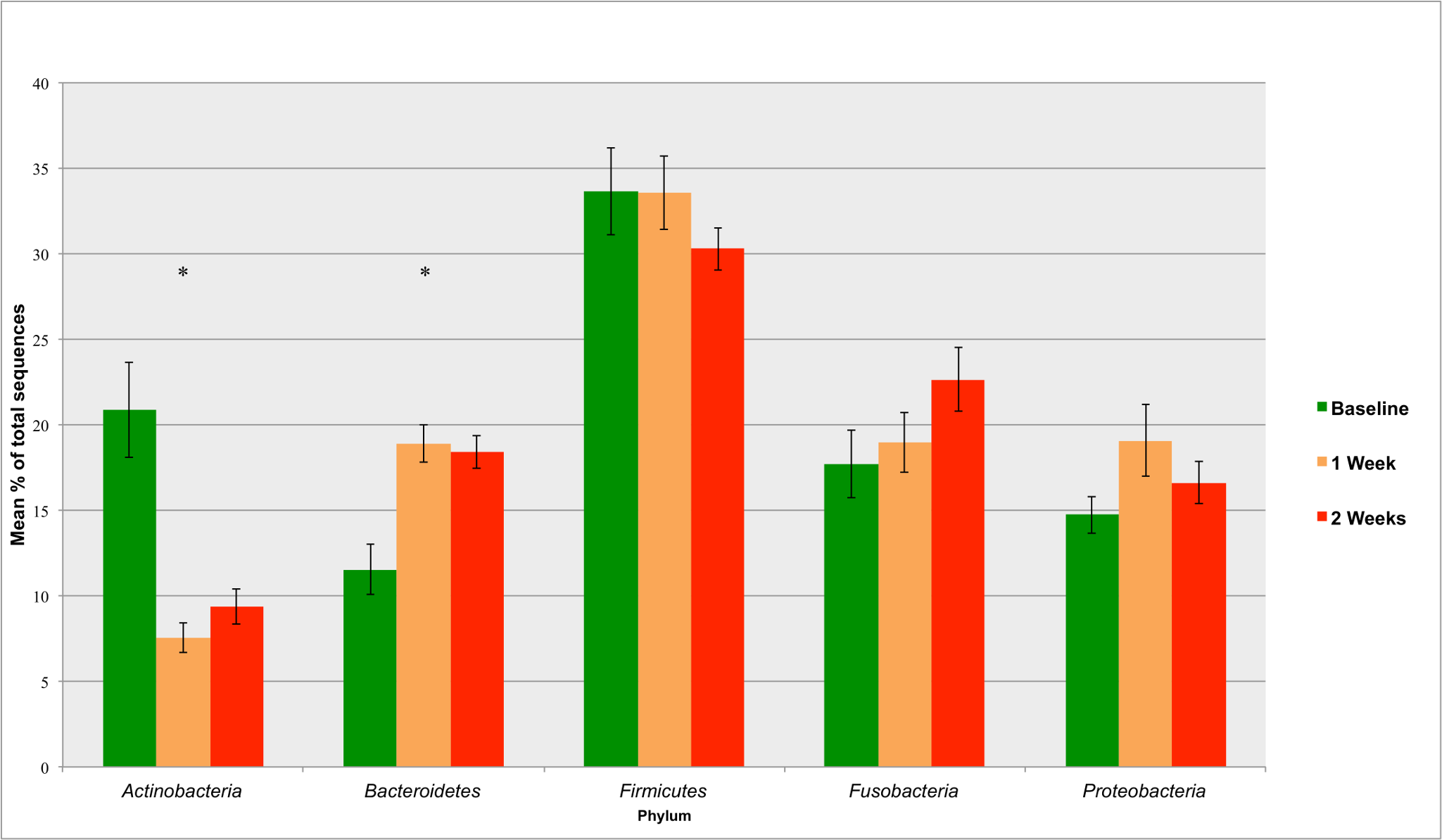

Supplement: Figure S6 — Relative abundances of the predominant phyla during the induction of experimental gingivitis. Histogram chart comparing the mean relative abundances of the predominant phyla at the different time points of experimental gingivitis. Statistically significant differences as indicated by two-sample t-tests are highlighted with an * and error bars shown are the standard error of the mean (SEM). (TIF) [file pone.0071227.s006.tif]

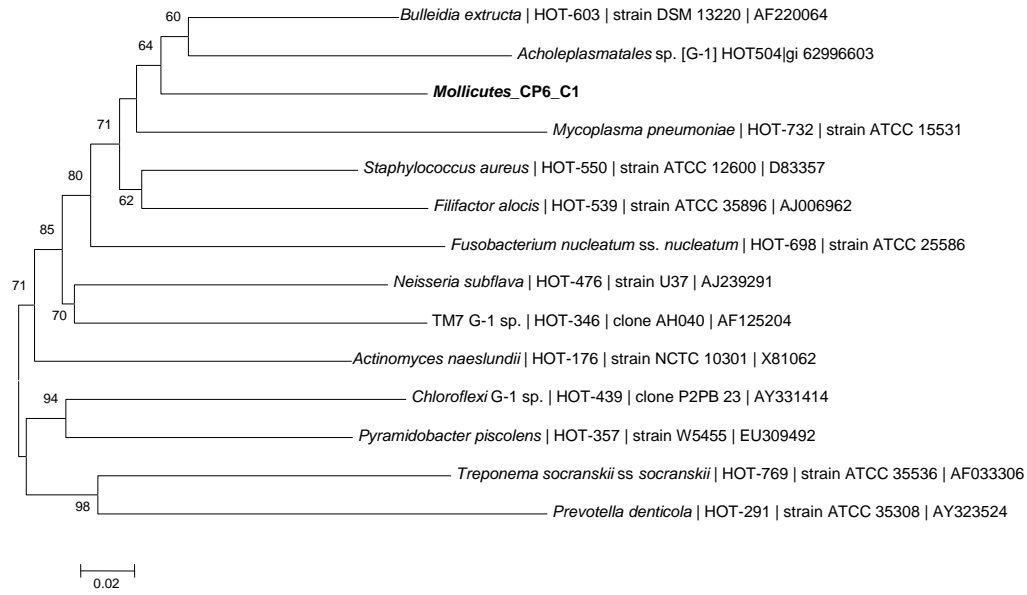

Supplement: Figure S7 — Phylogenetic tree based on 16S rRNA gene comparisons showing the relationship between Mollicutes_CP6_C1, members of the Firmicutes phylum and other phyla found in the oral cavity. The tree was constructed using the neighbor-joining method from a distance matrix constructed from aligned sequences using the Jukes-Cantor correction. Numbers represent bootstrap values for each branch based on data from 500 trees. Scale bars show the number of nucleotide substitutions per site. (PDF) [file pone.0071227.s007.pdf]

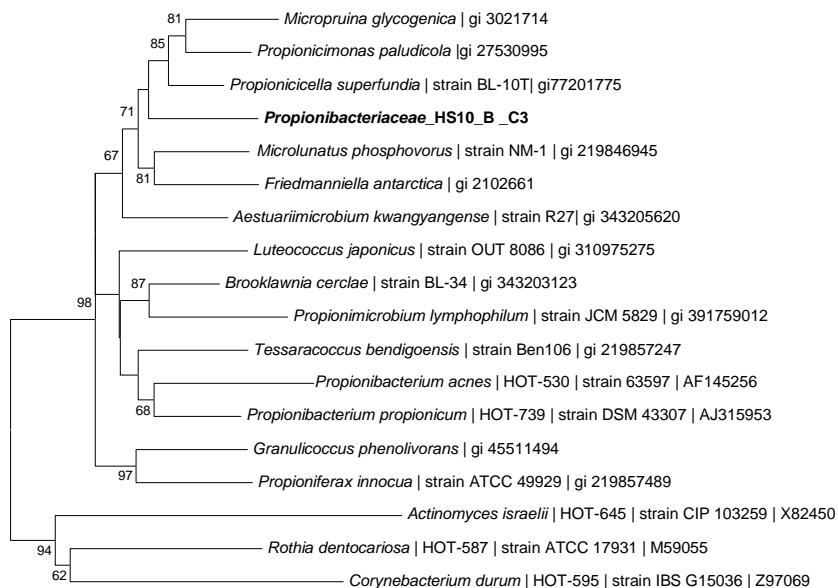

0.01

Supplement: Figure S8 — Phylogenetic tree based on 16S rRNA gene comparisons showing the relationship between Propionibacteriaceae HS10_B_C3 and members of the phylum Actinobacteria. The tree was constructed using the neighbor-joining method from a distance matrix constructed from aligned sequences using the Jukes-Cantor correction. Numbers represent bootstrap values for each branch based on data from 500 trees. Scale bars show the number of nucleotide substitutions per site. (PDF) [file pone.0071227.s008.pdf]

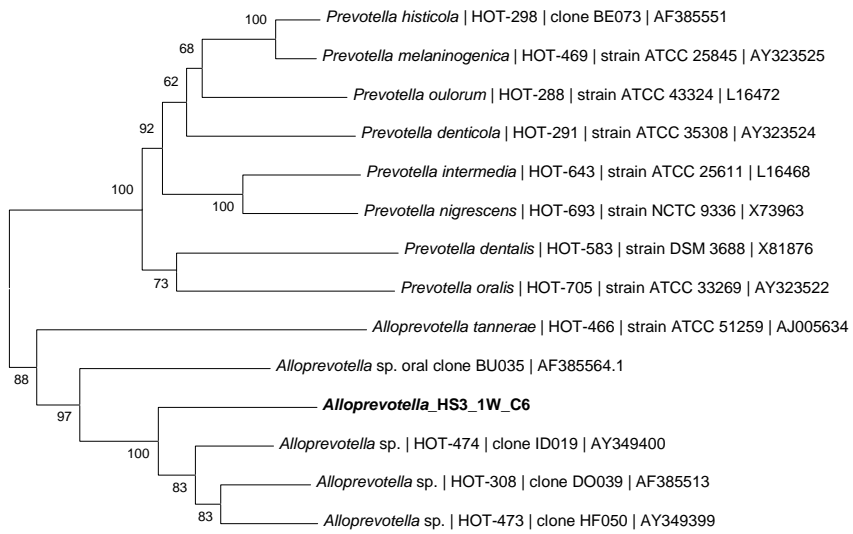

0.02

Supplement: Figure S9 — Phylogenetic tree based on 16S rRNA gene comparisons showing the relationship between, Alloprevotella_HS3_1W_C6 and members of the genera Alloprevotella and Prevotella. The tree was constructed using the neighbor-joining method from a distance matrix constructed from aligned sequences using the Jukes-Cantor correction. Numbers represent bootstrap values for each branch based on data from 500 trees. Scale bars show the number of nucleotide substitutions per site. (PDF) [file pone.0071227.s009.pdf]

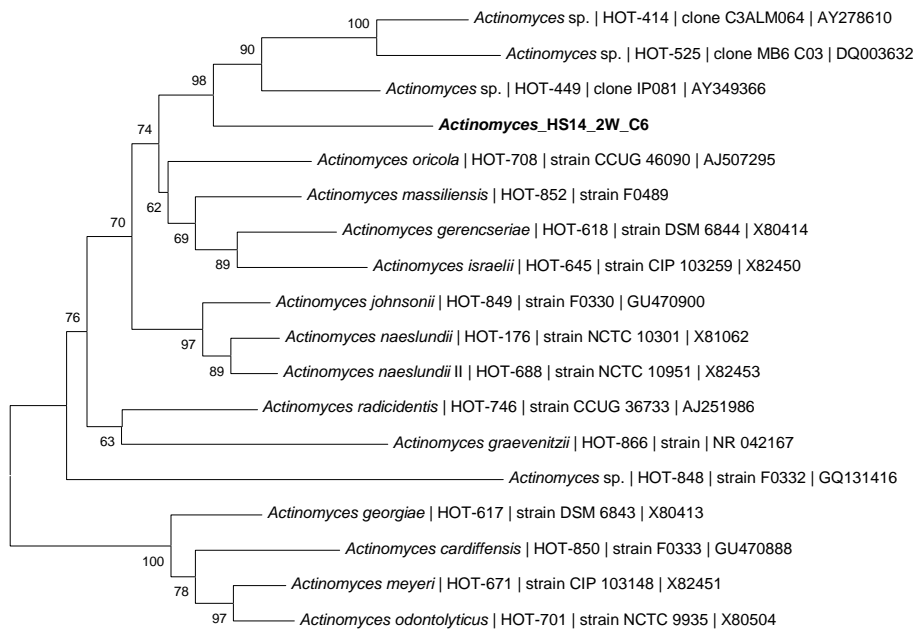

0.01

Supplement: Figure S10 — Phylogenetic tree based on 16S rRNA gene comparisons showing the relationship between, Actinomyces_HS14_2W_C6 and members of the genus Actinomyces. The tree was constructed using the neighbor-joining method from a distance matrix constructed from aligned sequences using the Jukes-Cantor correction. Numbers represent bootstrap values for each branch based on data from 500 trees. Scale bars show the number of nucleotide substitutions per site. (PDF) [file pone.0071227.s010.pdf]

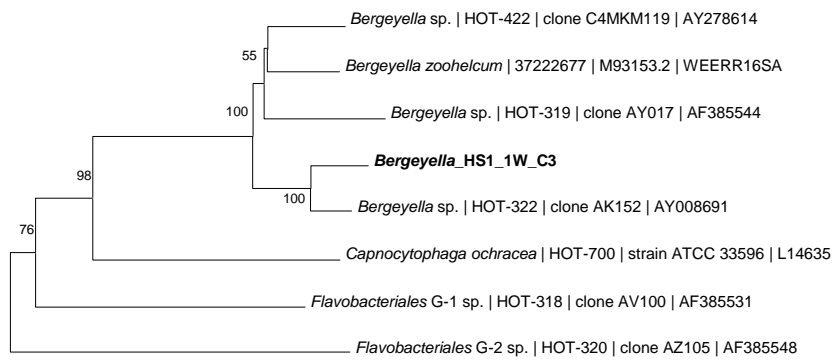

0.02

Supplement: Figure S11 — Phylogenetic tree based on 16S rRNA gene comparisons showing the relationship between Bergeyella_HS1_1W_C3, members of the genus Bergeyella and other members of the class Flavobacteria in the phylum Bacteroidetetes. The tree was constructed using the neighbor-joining method from a distance matrix constructed from aligned sequences using the Jukes-Cantor correction. Numbers represent bootstrap values for each branch based on data from 500 trees. Scale bars show the number of nucleotide substitutions per site. (PDF) [file pone.0071227.s011.pdf]

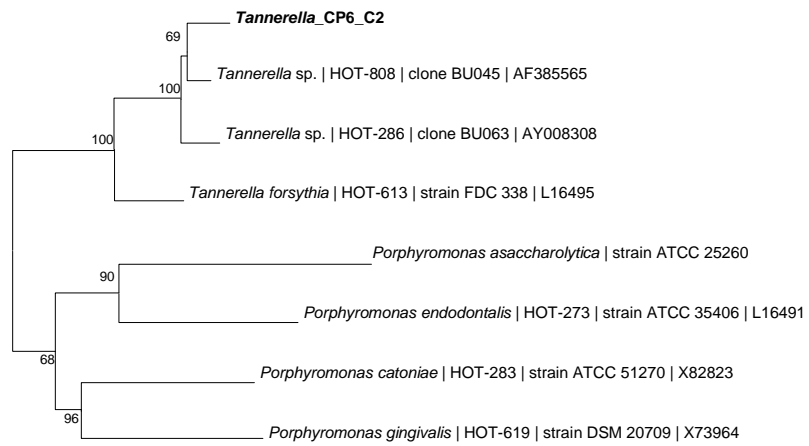

0.02

Supplement: Figure S12 — Phylogenetic tree based on 16S rRNA gene comparisons showing the relationship between Tannerella CP6_C2 and members of the genera Tannerella and Porphyromonas. The tree was constructed using the neighbor-joining method from a distance matrix constructed from aligned sequences using the Jukes-Cantor correction. Numbers represent bootstrap values for each branch based on data from 500 trees. Scale bars show the number of nucleotide substitutions per site. (PDF) [file pone.0071227.s012.pdf]

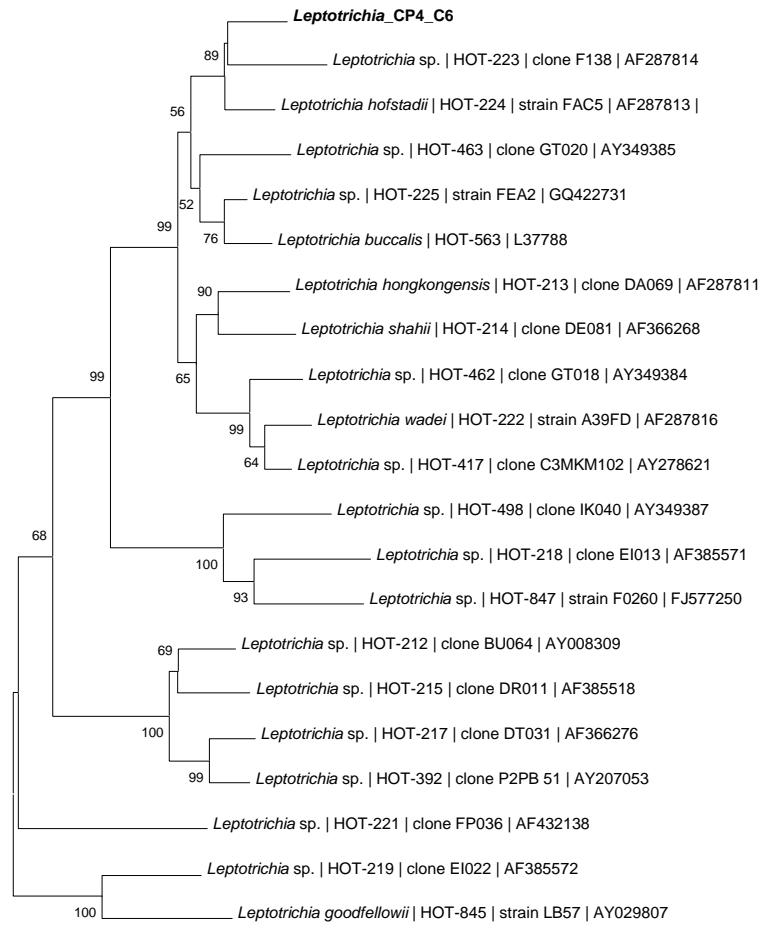

0.01

Supplement: Figure S13 — Phylogenetic tree based on 16S rRNA gene comparisons showing the relationship between Leptotrichia CP4_C6 and members of the genus Leptotrichia. The tree was constructed using the neighbor-joining method from a distance matrix constructed from aligned sequences using the Jukes-Cantor correction. Numbers represent bootstrap values for each branch based on data from 500 trees. Scale bars show the number of nucleotide substitutions per site. (PDF) [file pone.0071227.s013.pdf]

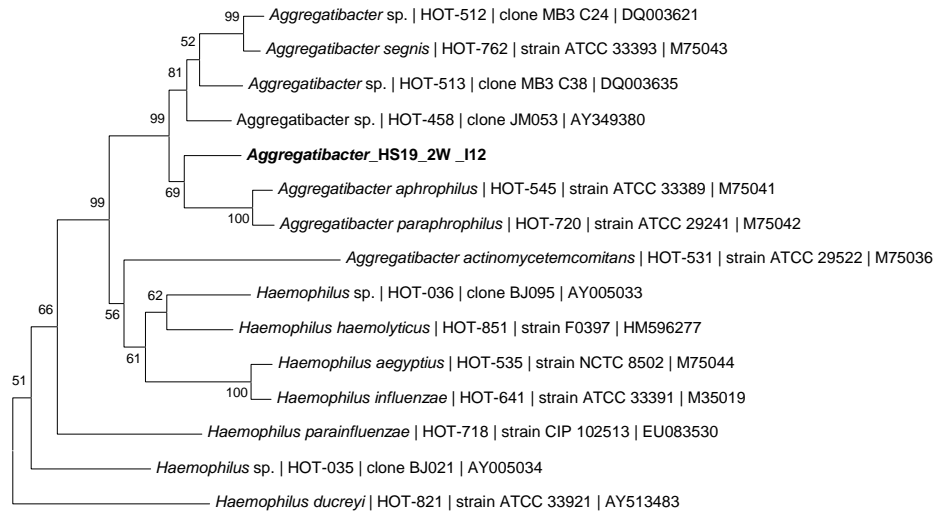

0.01

Supplement: Figure S14 — Phylogenetic tree based on 16S rRNA gene comparisons showing the relationship between Aggregatibacter_HS19_2W_I12 and members of the genera Aggregatibacter and Haemophilus. The tree was constructed using the neighbor-joining method from a distance matrix constructed from aligned sequences using the Jukes-Cantor correction. Numbers represent bootstrap values for each branch based on data from 500 trees. Scale bars show the number of nucleotide substitutions per site. (PDF) [file pone.0071227.s014.pdf]

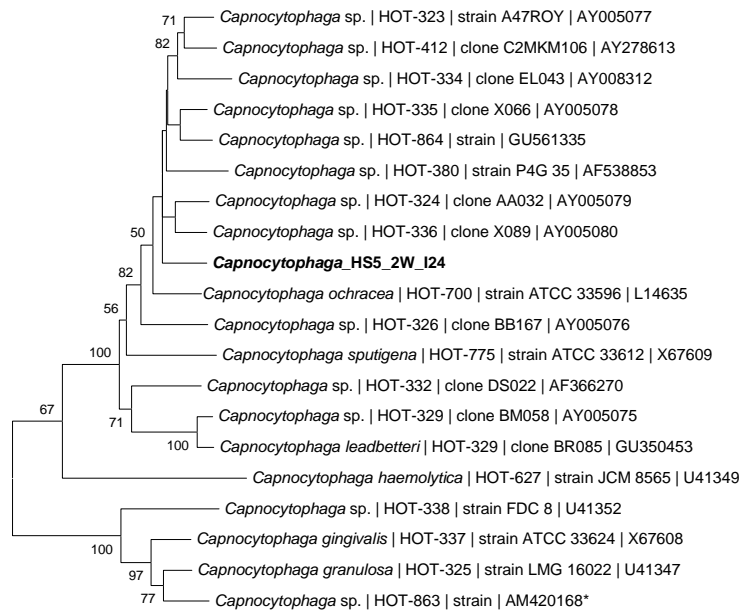

0.02

Supplement: Figure S15 — Phylogenetic tree based on 16S rRNA gene comparisons showing the relationship between Capnocytophaga_HS5_2W_I24 and members of the genus Capnocytophaga. The tree was constructed using the neighbor-joining method from a distance matrix constructed from aligned sequences using the Jukes-Cantor correction. Numbers represent bootstrap values for each branch based on data from 500 trees. Scale bars show the number of nucleotide substitutions per site. (PDF) [file pone.0071227.s015.pdf]
