# Supplementary material for: Bacterial Community Development in Experimental Gingivitis
Source: PLoS One. 2013 Aug 14;8(8):e71227. doi: 10.1371/journal.pone.0071227 (PMC3743832; doi:10.1371/journal.pone.0071227)
Supplement: Table S1 — Alpha diversity of plaque samples. (DOC) [file pone.0071227.s016.doc]

| **Sample ID** | **No. of seqs** | **No. of observed OTUs** | **Good’s coverage (%)** | **Chao1 total OTU richness estimate** | **CatchAll total OTU richness estimate** | **Simpson’s inverse diversity index** | **Simpson’s evenness** |
| --- | --- | --- | --- | --- | --- | --- | --- |
| HS1_B | 3061 | 326 | 95.6 | 508 | 738 | 37.7 | 0.12 |
| HS1_1W | 4663 | 349 | 97.2 | 535 | 1034 | 46.1 | 0.13 |
| HS1_2W | 3303 | 283 | 97.1 | 455 | 881 | 48.6 | 0.17 |
| HS2_B | 3479 | 201 | 97.2 | 434 | 435 | 14.5 | 0.07 |
| HS3_B | 4722 | 243 | 97.4 | 543 | 780 | 8.3 | 0.03 |
| HS3_1W | 4526 | 332 | 97.1 | 530 | 926 | 30.9 | 0.09 |
| HS3_2W | 5104 | 361 | 97.4 | 529 | 838 | 40.8 | 0.11 |
| HS4_B | 4978 | 301 | 97.4 | 509 | 751 | 17.3 | 0.06 |
| HS41_W | 5542 | 299 | 98.0 | 457 | 1690 | 22.6 | 0.08 |
| HS4_2W | 5271 | 350 | 97.8 | 469 | 659 | 39.4 | 0.11 |
| HS5_B | 3210 | 316 | 96.0 | 502 | 688 | 32.5 | 0.10 |
| HS5_1W | 3464 | 281 | 96.4 | 484 | 610 | 37.8 | 0.13 |
| HS5_2W | 3075 | 327 | 95.9 | 491 | 676 | 43.9 | 0.13 |
| HS6_B | 3176 | 250 | 96.7 | 384 | 547 | 18.9 | 0.08 |
| HS6_1W | 2697 | 258 | 96.2 | 442 | 876 | 42.7 | 0.17 |
| HS6_2W | 3559 | 329 | 96.9 | 478 | 689 | 71.2 | 0.22 |
| HS7_B | 3151 | 226 | 96.7 | 388 | 612 | 22.2 | 0.10 |
| HS7_1W | 2698 | 253 | 96.3 | 374 | 638 | 33.3 | 0.13 |
| HS7_2W | 3603 | 300 | 97.2 | 407 | 541 | 48.4 | 0.16 |
| HS8_B | 3549 | 256 | 96.7 | 478 | 474 | 25.2 | 0.10 |
| HS8_1W | 4439 | 268 | 97.5 | 466 | 499 | 11.6 | 0.04 |
| HS8_2W | 2941 | 287 | 96.4 | 431 | 470 | 47.9 | 0.17 |
| HS9_B | 3220 | 354 | 95.7 | 520 | 804 | 46.1 | 0.13 |
| HS9_1W | 3231 | 311 | 96.3 | 446 | 595 | 37.3 | 0.12 |
| HS9_2W | 3292 | 325 | 96.8 | 419 | 549 | 59.2 | 0.18 |
| HS10_B | 3698 | 369 | 96.1 | 570 | 817 | 52.7 | 0.14 |
| HS10_1W | 3135 | 339 | 95.5 | 586 | 884 | 50.3 | 0.15 |
| HS10_2W | 3573 | 386 | 96.1 | 557 | 668 | 64.5 | 0.17 |
| HS11_B | 3294 | 297 | 96.6 | 483 | 603 | 44.3 | 0.15 |
| HS11_1W | 3313 | 344 | 95.9 | 544 | 978 | 49.2 | 0.14 |
| HS11_2W | 2909 | 298 | 96.3 | 433 | 675 | 46.1 | 0.15 |
| HS12_B | 4121 | 376 | 96.2 | 577 | 807 | 30.3 | 0.08 |
| HS12_1W | 3276 | 312 | 96.0 | 469 | 725 | 17.4 | 0.06 |
| HS12_2W | 2876 | 302 | 96.0 | 445 | 507 | 34.2 | 0.11 |
| HS13_B | 3536 | 280 | 96.6 | 487 | 550 | 23.3 | 0.08 |
| HS13_1W | 3106 | 250 | 96.6 | 392 | 536 | 24.8 | 0.10 |
| HS13_2W | 4159 | 303 | 97.1 | 482 | 1161 | 18.1 | 0.06 |
| HS14_B | 4162 | 344 | 96.4 | 592 | 764 | 36.6 | 0.11 |
| HS14_1W | 3487 | 271 | 97.2 | 412 | 776 | 37.3 | 0.14 |
| HS14_2W | 3606 | 254 | 97.4 | 397 | 438 | 35.1 | 0.14 |
| HS15_B | 4350 | 380 | 96.8 | 557 | 649 | 45.7 | 0.12 |
| HS15_1W | 3550 | 317 | 96.7 | 443 | 585 | 48.3 | 0.15 |
| HS15_2W | 3997 | 385 | 96.4 | 576 | 730 | 59.4 | 0.15 |
| HS16_B | 4343 | 302 | 97.3 | 510 | 531 | 38.0 | 0.13 |
| HS16_1W | 5746 | 290 | 97.9 | 532 | 563 | 10.2 | 0.04 |
| HS16_2W | 4469 | 346 | 97.2 | 498 | 668 | 49.7 | 0.14 |
| HS17_B | 4049 | 246 | 97.6 | 369 | 468 | 16.7 | 0.07 |
| HS17_1W | 3357 | 209 | 97.9 | 293 | 522 | 31.1 | 0.15 |
| HS17_2W | 4141 | 261 | 97.6 | 426 | 459 | 52.7 | 0.20 |
| HS18_B | 3363 | 249 | 97.0 | 423 | 522 | 23.4 | 0.09 |
| HS18_1W | 2973 | 259 | 96.6 | 406 | 553 | 41.5 | 0.16 |
| HS18_2W | 3545 | 273 | 96.5 | 491 | 866 | 27.0 | 0.10 |
| HS19_B | 5811 | 383 | 97.4 | 625 | 834 | 32.2 | 0.08 |
| HS19_1W | 3885 | 287 | 97.2 | 437 | 591 | 34.6 | 0.12 |
| HS19_2W | 3896 | 348 | 97.0 | 496 | 525 | 63.5 | 0.18 |
| HS20_B | 3732 | 262 | 97.2 | 433 | 817 | 32.9 | 0.13 |
| HS20_1W | 3956 | 281 | 97.1 | 448 | 829 | 20.2 | 0.07 |
| HS20_2W | 4294 | 304 | 97.2 | 461 | 731 | 33.0 | 0.11 |
| CP1 | 3803 | 358 | 95.8 | 603 | 928 | 21.3 | 0.06 |
| CP2 | 3128 | 373 | 95.4 | 586 | 977 | 71.7 | 0.19 |
| CP3 | 4001 | 330 | 96.6 | 554 | 1021 | 47.6 | 0.14 |
| CP4 | 3536 | 294 | 97.0 | 418 | 530 | 29.9 | 0.10 |
| CP5 | 4289 | 386 | 96.5 | 581 | 1006 | 54.1 | 0.14 |
| CP6 | 3494 | 394 | 95.9 | 557 | 821 | 64.2 | 0.16 |
| CP7 | 3472 | 367 | 96.0 | 583 | 743 | 47.0 | 0.13 |
| CP7_Sub | 3839 | 309 | 97.3 | 418 | 631 | 50.9 | 0.16 |
| CP8 | 5797 | 308 | 97.7 | 503 | 869 | 14.8 | 0.05 |
| CP8_Sub | 3842 | 268 | 97.1 | 438 | 1059 | 11.5 | 0.04 |
| CP9 | 3801 | 287 | 96.7 | 516 | 620 | 24.7 | 0.09 |
| CP9_Sub | 4492 | 350 | 96.9 | 541 | 653 | 42.0 | 0.12 |
| CP10 | 3288 | 330 | 95.9 | 537 | 830 | 45.0 | 0.14 |
| CP10_Sub | 4859 | 394 | 96.8 | 634 | 858 | 56.4 | 0.14 |
| CP11 | 5304 | 241 | 98.4 | 345 | 585 | 17.9 | 0.07 |
| CP11_Sub | 4405 | 250 | 98.1 | 356 | 349 | 29.0 | 0.12 |
| CP12 | 5201 | 355 | 97.0 | 609 | 1204 | 23.8 | 0.07 |
| CP12_Sub | 4782 | 345 | 97.3 | 516 | 566 | 13.4 | 0.04 |
| CP13 | 2478 | 89 | 98.7 | 130 | 200 | 13.2 | 0.15 |
| CP13_Sub | 2810 | 176 | 97.9 | 269 | 322 | 15.8 | 0.09 |
| CP14 | 2156 | 136 | 97.5 | 225 | 298 | 12.9 | 0.09 |
| CP14_Sub | 3017 | 186 | 97.7 | 290 | 290 | 18.5 | 0.10 |
| CP15 | 2813 | 260 | 96.4 | 375 | 599 | 32.5 | 0.12 |
| CP15_Sub | 2847 | 271 | 96.8 | 366 | 505 | 48.6 | 0.18 |
| CP16 | 3956 | 211 | 98.2 | 357 | 390 | 22.8 | 0.11 |
| CP16_Sub | 3657 | 201 | 98.1 | 325 | 1152 | 15.3 | 0.08 |
| CP17 | 2651 | 216 | 97.7 | 266 | 350 | 26.8 | 0.12 |
| CP17_Sub | 3112 | 158 | 98.4 | 203 | 240 | 9.2 | 0.06 |
| CP18 | 3899 | 331 | 97.3 | 455 | 637 | 39.3 | 0.12 |
| CP18_Sub | 3113 | 301 | 96.9 | 400 | 573 | 51.1 | 0.17 |
| CP19 | 2875 | 235 | 96.4 | 478 | 1039 | 41.3 | 0.18 |
| CP19_Sub | 3310 | 250 | 97.4 | 371 | 435 | 36.9 | 0.15 |
| CP20 | 2856 | 231 | 97.1 | 360 | 431 | 42.6 | 0.18 |
| CP20_Sub[[1]](#endnote-2) | 3722 | 242 | 97.4 | 376 | 558 | 32.1 | 0.13 |

1. HS = Healthy subject, 1W = 1 week, 2W = 2 weeks, CP = Chronic periodontitis patient, Sub = subgingival plaque, Numbers indicate subject or patient number. [↑](#endnote-ref-2)
