# Supplementary material for: Bacterial Community Development in Experimental Gingivitis
Source: PLoS One. 2013 Aug 14;8(8):e71227. doi: 10.1371/journal.pone.0071227 (PMC3743832; doi:10.1371/journal.pone.0071227)
Supplement: Table S2 — OTUs associated with time points of experimental gingivitis. OTUs were associated with time points of experimental gingivitis using Multivariate Association with Linear Models (MaAsLin). OTUs are ranked according to their P value. OTUs listed have P values <0.05. (DOC) [file pone.0071227.s017.doc]

| **OTU / Taxon** | **Time point** | **Coefficient** | ***P* value** | ***Q* value** |
| --- | --- | --- | --- | --- |
| Otu0037 *Lautropia* sp. HOTA94 | 2 weeks | 0.073948124 | 0.00011871 | 0.010565229 |
| Otu0118 *Campylobacter showae* | 2 weeks | 0.050803532 | 0.000904104 | 0.040232625 |
| Otu0447 *Prevotella oulorum* | 2 weeks | 0.03167685 | 0.001028981 | 0.030526436 |
| Otu0118 *Campylobacter showae* | 1 week | 0.049116269 | 0.001332742 | 0.029653513 |
| Otu0002 *Rothia dentocariosa* | 2 weeks | -0.072626168 | 0.001895256 | 0.033735555 |
| Otu0582 *Porphyromonas catoniae* | 2 weeks | 0.04147979 | 0.002073785 | 0.030761148 |
| Otu0020 *Fusobacterium nucleatum* subsp.*polymorphum* | 2 weeks | 0.061051369 | 0.002820397 | 0.03585933 |
| Otu0045 *Actinobaculum* sp. HOT183 | 1 week | -0.021679771 | 0.005100867 | 0.05674714 |
| Otu0199 *Lachnospiraceae* sp. [G-2] HOT100 | 2 weeks | 0.034600546 | 0.008671769 | 0.085754161 |
| Otu0008 *Streptococcus mitis*/ HOT064/ HOT423/ HOTA95/ HOTE14 | 2 weeks | -0.073260824 | 0.00957342 | 0.085203435 |
| Otu0113 *Propionibacterium* sp. HOT194 | 1 week | -0.032815443 | 0.009679339 | 0.078314648 |
| Otu0189 *Tannerella* sp. HOT286 | 2 weeks | 0.046722744 | 0.009723998 | 0.072119653 |
| Otu0199 *Lachnospiraceae* sp. [G-2] HOT100 | 1 week | 0.033706285 | 0.010737132 | 0.073508061 |
| Otu0037 *Lautropia* sp. HOTA94 | 1 week | 0.048161992 | 0.012769668 | 0.081178606 |
| Otu0021 *Fusobacterium nucleatum* subsp. *polymorphum* | 1 week | 0.032618934 | 0.01351712 | 0.080201578 |
| Otu0045 *Actinobaculum* sp. HOT183 | 2 weeks | -0.019217291 | 0.013950516 | 0.077599748 |
| Otu0195 *Leptotrichia* sp. HOT212 | 2 weeks | 0.027206623 | 0.014672469 | 0.076814692 |
| Otu0002 *Rothia dentocariosa* | 1 week | -0.055133036 | 0.020789757 | 0.102793797 |
| Otu0027 *Corynebacterium durum* | 1 week | -0.032343834 | 0.021311249 | 0.099826375 |
| Otu0182 *Leptotrichia* sp. HOT417 | 2 weeks | 0.039218631 | 0.022517111 | 0.100201143 |
| Otu0629 *Selenomonas dianae* | 2 weeks | 0.033516948 | 0.022520435 | 0.095443747 |
| Otu0128 *Capnocytophaga sputigena* | 1 week | 0.044737563 | 0.023019741 | 0.093125317 |
| Otu0141 *Actinomyces naeslundii* | 1 week | -0.036405586 | 0.025881979 | 0.100152006 |
| Otu0020 *Fusobacterium nucleatum* subsp.*polymorphum* | 1 week | 0.045411958 | 0.030681591 | 0.113777566 |
| Otu0066 *Capnocytophaga leadbetteri* | 1 week | 0.053924512 | 0.035581727 | 0.126670947 |
| Otu0364 *Leptotrichia hongkongensis* | 2 weeks | -0.046228529 | 0.036615693 | 0.125338333 |
| Otu0582 *Porphyromonas catoniae* | 1 week | 0.028729504 | 0.039545082 | 0.130352308 |
| Otu0084 *Gemella morbillorum* | 2 weeks | 0.047656497 | 0.040767131 | 0.129581236 |
| Otu0113 *Propionibacterium* sp. HOT194 | 2 weeks | -0.026083639 | 0.046482656 | 0.14265367 |
| Otu0021 *Fusobacterium nucleatum* subsp. *polymorphum* | 2 weeks | 0.026826325 | 0.048688265 | 0.144441852 |
| Otu0189 *Tannerella* sp. HOT286 | 1 week | 0.036820748 | 0.048931194 | 0.140479879 |
