# Supplementary material for: Bacterial Community Development in Experimental Gingivitis
Source: PLoS One. 2013 Aug 14;8(8):e71227. doi: 10.1371/journal.pone.0071227 (PMC3743832; doi:10.1371/journal.pone.0071227)
Supplement: Table S3 — OTUs associated with bleeding on probing scores. OTUs were associated with BoP using Multivariate Association with Linear Models (MaAsLin). OTUs are ranked according to their P value. OTUs listed have P values <0.05. (DOC) [file pone.0071227.s018.doc]

| **OTU / Taxon** | **Coefficient** | ***P* value** | ***Q* value** |
| --- | --- | --- | --- |
| Otu0037 *Lautropia* sp. HOTA94 | 0.002220345 | 1.60E-05 | 0.003206 |
| Otu0199 *Lachnospiraceae* sp. [G-2] HOT100 | 0.001067424 | 0.000319262 | 0.031926 |
| Otu0447 *Prevotella oulorum* | 0.000933123 | 0.000477674 | 0.031845 |
| Otu0002 *Rothia dentocariosa* | -0.002473849 | 0.000993964 | 0.049698 |
| Otu0020 *Fusobacterium nucleatum* subsp. *polymorphum* | 0.002011651 | 0.001161698 | 0.046468 |
| Otu0582 *Porphyromonas catoniae* | 0.001229655 | 0.001899077 | 0.063303 |
| Otu0118 *Campylobacter showae* | 0.001422896 | 0.002739583 | 0.078274 |
| Otu0223 *Solobacterium moorei* | 0.000814783 | 0.004457756 | 0.111444 |
| Otu0182 *Leptotrichia* sp. HOT417 | 0.001251437 | 0.005561952 | 0.123599 |
| Otu0014 *Haemophilus parainfluenzae* | -0.001623441 | 0.010888484 | 0.21777 |
| Otu0027 *Corynebacterium durum* | -0.001040609 | 0.012572451 | 0.22859 |
| Otu0189 *Tannerella* sp. HOT286 | 0.00122397 | 0.014983566 | 0.249726 |
| Otu0045 *Actinobaculum* sp. HOT183 | -0.000600708 | 0.018026678 | 0.277334 |
| Otu0112 *Leptotrichia buccalis* | 0.002021963 | 0.019507108 | 0.278673 |
| Otu0303 *Parvimonas micra* | 0.000436195 | 0.020234447 | 0.269793 |
| Otu0021 *Fusobacterium nucleatum* subsp. *polymorphum* | 0.00079256 | 0.020487774 | 0.256097 |
| Otu0008 *Streptococcus mitis* / HOT064/ HOT423/ HOTA95/ HOTE14 | -0.001968185 | 0.020746311 | 0.244074 |
| Otu0040 *Corynebacterium durum* | -0.000908473 | 0.022691505 | 0.252128 |
| Otu0364 *Leptotrichia hongkongensis* | -0.001423323 | 0.031204461 | 0.328468 |
| Otu0066 *Capnocytophaga leadbetteri* | 0.001169352 | 0.037508802 | 0.375088 |
| Otu0033 *Actinomyces* sp. HOT169 | -0.001238874 | 0.041586298 | 0.39606 |
