# Supplementary material for: Bacterial Community Development in Experimental Gingivitis
Source: PLoS One. 2013 Aug 14;8(8):e71227. doi: 10.1371/journal.pone.0071227 (PMC3743832; doi:10.1371/journal.pone.0071227)
Supplement: Table S4 — Prevalence of novel taxa in the experimental gingivitis and chronic periodontitis cohorts. (DOC) [file pone.0071227.s019.doc]

| **Assigned HOMD taxon name** | **No. of samples in which phylotype was detected (%)** | | | | |
| --- | --- | --- | --- | --- | --- |
|  | **Experimental gingivitis** | | | **Chronic periodontitis** | |
|  | **Baseline (*n*=20)** | **1 week**  **(*n*=19)** | **2 weeks (*n*=19)** | **Superficial plaque (*n*=20)** | **Subgingival plaque (*n*=14)** |
| *Mollicutes* sp. HOT906 | 0 (0) | 0 (0) | 0 (0) | 2 (10) | 1 (7) |
| *Propionibacterium* sp. HOT915 | 3 (15) | 0 (0) | 2 (11) | 1 (5) | 0 (0) |
| *Alloprevotella* sp. HOT914 | 0 (0) | 4 (21) | 4 (21) | 1 (5) | 1 (7) |
| *Actinomyces* sp. HOT897 | 7 (35) | 5 (26) | 8 (42) | 7 (35) | 2 (14) |
| *Bergeyella* sp. HOT900 | 2 (10) | 4 (21) | 2 (11) | 2 (10) | 2 (14) |
| *Tannerella* sp. HOT916 | 2 (10) | 1 (5) | 2 (11) | 6 (30) | 3 (21) |
| *Leptotrichia* sp. HOT909 | 1 (5) | 2 (11) | 2 (11) | 1 (5) | 0 (0) |
| *Aggregatibacter* sp. HOT898 | 1 (5) | 4 (21) | 4 (21) | 1 (5) | 0 (0) |
| *Capnocytophaga* sp. HOT903 | 1 (5) | 0 (0) | 1 (5) | 0 (0) | 0 (0) |
